# Identification and mapping of QTLs for late blight resistance in the wild tomato (*Solanum pimpinellifolium*) accession PI 270442 via selective genotyping

**DOI:** 10.3389/fpls.2024.1482241

**Published:** 2024-11-15

**Authors:** Matthew T. Sullenberger, Majid R. Foolad

**Affiliations:** Department of Plant Science and the Intercollege Graduate Degree Program in Plant Biology, The Pennsylvania State University, University, Park, PA, United States

**Keywords:** genotyping-by-sequencing, *Phytophthora infestans*, reduced representation library, SNP markers, *Solanum lycopersicum*, trait-based analysis, disease resistance

## Abstract

Late blight (LB), caused by the oomycete *Phytophthora infestans*, is one of the most devastating diseases of the cultivated tomato (*Solanum lycopersicum*) worldwide. Attempts to control the disease through fungicide applications are becoming less effective, as new and aggressive genotypes of the pathogen emerge. Further, some new *P. infestans* genotypes overcome the currently available resistance in tomato, necessitating the identification, characterization, and utilization of new sources of host resistance. In the present study, to detect QTLs underlying LB resistance in a recently-identified LB-resistant *S. pimpinellifolium* accession (PI 270442), an F_2_ population (n = 1,175) of a cross between PI 270442 and LB-susceptible tomato breeding line Fla. 8059 was screened for LB resistance and subjected to selective genotyping. A total of 19,839 single-nucleotide polymorphisms (SNPs) were identified from reduced representation libraries (RRLs) constructed from the parents, of which 212 were used to build a genetic linkage map and locate QTLs. Ten LB-resistance QTLs were identified in PI 270442 on chromosomes 1, 2, 5, 6, 10, 11 and 12, of which those on chromosomes 6, 10 and 11 were the strongest and co-localized with previously-reported LB-resistance QTLs. Genomic locations of the QTLs were compared with the tomato physical map, which resulted in the identification of several candidate genes that might be underpinning the LB resistance in PI 270442. The identified QTL-linked markers can be used in breeding programs to transfer resistance from PI 270442 into the cultivated tomato via marker-assisted breeding and to develop near-isogenic lines for fine mapping of the QTLs.

## 1 Introduction

The cultivated tomato, *Solanum lycopersicum* L., was valued at $100.5 billion in 2021 (FAO, 2023), making it the most valuable vegetable crop in the world. However, plant diseases create significant losses in yield and quality of the tomato, in addition to the cost associated with disease management (Foolad, 2007). Late blight (LB), caused by the oomycete *Phytophthora infestans* (Mont.) de Bary, is one of the most detrimental and costly diseases of the cultivated tomato and potato (*S. tuberosum* L.) worldwide (Fry and Goodwin, 1997). The pathogen can asexually reproduce within one week and potentially destroy an entire crop in 7 – 10 days when conditions are favorable for disease progression (Fry and Goodwin, 1997; Nowicki et al., 2012; Fry et al., 2013). Currently, the most common approach to managing LB in tomato is preventative and involves weekly fungicide applications, leading to economic and environmental repercussions (Fry and Goodwin, 1997). Additionally, some *P. infestans* isolates have developed either resistance (e.g. clonal lineages US-6, US-7, US-8 and US-11) or intermediate resistance (e.g. US-23 and US-24) to metalaxyl, one of the most effective systemic fungicides against the pathogen (Matson et al., 2015), leaving the fungicide largely ineffective (Chowdappa et al., 2015).

Another approach to managing the disease is through plant host resistance. In potato, most current cultivars carry one or more LB-resistance genes (Bradshaw and Ramsay, 2005; Tiwari et al., 2013), with at least one resistance gene or quantitative trait locus (QTL) mapped to every chromosome (Tiwari et al., 2013). These include *R* genes found in potato wild species *S. demissum* Lindl., *S. bulbocastanum* Dunal, *S. berthaultii* Hawkes, *S. mochiquense* Ochoa, *S. phureja* Juz. & Bukasov., *S. pinnatisectum* Dunal., and *S. ruiz-ceballosii* Cárd (Ewing et al., 2000; Song et al., 2003; van der Vossen et al., 2003; Smilde et al., 2005; Rauscher et al., 2006; Sliwka et al., 2006; Brylińska et al., 2015; Jo et al., 2015). To date, all cloned LB-resistance genes in potato encode NBS-LRR proteins (Rodewald and Trognitz, 2013), suggesting that this gene family is a major contributor to vertical resistance to potato LB disease (Vleeshouwers et al., 2011). This raises the possibility of identifying *R* genes in the wild species of tomato, a close relative of the potato. There may also be potential in identifying LB-resistance genes in tomato from *R-gene* homologs in potato.

Limited genetic diversity exists within the cultivated species of tomato, and only a few LB-resistance genes or QTLs have been identified in the cultivated species. However, the vast majority of genetic diversity (95%) across all 13 tomato species is estimated to exist within the related wild species, including many beneficial horticultural traits such as resistance/tolerance to biotic and abiotic stresses (Miller and Tanksley, 1990; Hu et al., 2012). Many of the desirable traits have been identified within the wild tomato species *S. pimpinellifolium*, *S. habrochaites*, and *S. peruvianum* (Foolad, 2007; Foolad et al., 2008; Solankey et al., 2017), with the most useful LB-resistance genes originating from the closest wild species *S. pimpinellifolium* (Foolad et al., 2008). The first LB-resistance gene identified in tomato, *Ph-1*, is located on chromosome 7 in *S. pimpinellifolium* accessions West Virginia 19 and West Virginia 731 and confers resistance against only race T_0_ of *P. infestans* (Bonde and Murphy, 1952), which has been displaced by race T_1_, rendering *Ph-1* resistance ineffective (Conover and Walter, 1953; USABlight, 2015; USDA, 2015). The second LB-resistance gene in tomato, *Ph-2*, expressing incomplete dominance and conferring only partial resistance against *P. infestans*, was originally identified in *S. pimpinellifolium* accession West Virginia 700 (Gallegly and Marvel, 1955; Gallegly, 1960). Subsequently, *Ph-2* was mapped to the long arm of chromosome 10 (Moreau et al., 1998) and fine mapped to a 141-kb genomic region on the distal end of the chromosome (Zhi et al., 2021). *Ph-2* has been deployed in several fresh market (FM) and processing (PROC) tomato cultivars, providing partial resistance to various *P. infestans* US clonal lineages, including US-13, US-14, and US-23 (Goodwin et al., 1995; Black et al., 1996; Moreau et al., 1998; Foolad et al., 2014). The third and strongest commercially-available LB-resistance gene in tomato is *Ph-3*, which was originally identified in *S. pimpinellifolium* accession L3708 (Black et al., 1996) and mapped to the long arm of tomato chromosome 9 (Black et al., 1996; Chunwongse et al., 2002). This gene was recently fine mapped to a 26-kb region, cloned, and characterized; it encodes a CC-NBS-LRR protein (Zhang et al., 2014). Tomato breeding lines and hybrid cultivars containing *Ph-3* alone exhibit good resistance against several *P. infestans* clonal lineages, including US-13, US-14, and US-23; however, severe LB symptoms have been observed on such genotypes in various tomato field trials around the world (Chunwongse et al., 2002; MR Foolad, *unpublished*, RG Gardner, *personal communication*). In contrast, stacking *Ph-2* and *Ph-3* offers the most effective and durable resistance against a broad range of *P. infestans* isolates/clonal lineages (Gardner and Panthee, 2010).

While breeding lines and hybrid cultivars with *Ph-2* and *Ph-3* combined exhibit the strongest resistance (Fry et al., 2013; Foolad et al., 2014; Hansen et al., 2014), under disease favorable conditions and high disease pressure such genotypes show some level of LB disease (MR Foolad, *unpublished*; RG Gardner, *personal communication*). It is, therefore, important to identify and characterize additional genetic sources of LB resistance in tomato and incorporate them into new breeding lines and hybrid cultivars. We previously screened 67 accessions of *S. pimpinellifolium* for LB resistance under field and greenhouse conditions, which resulted in the identification of strong LB resistance in 12 accessions (Foolad et al., 2014; Gugino et al., 2014; Foolad et al., 2015). Here we explore the genetic basis of LB resistance in one of those 12 accessions, PI 270442.

Since the first release of the tomato genome sequence in 2012 (Consortium, 2012; Hosmani et al., 2019), more than 1,000 other tomato accessions and cultivars have been sequenced (SGN, 2019). Genetic mapping has become more accessible and common in tomato, leading to the construction of several high-density genetic linkage maps using SNP markers. For example, recently we reported a highly-saturated genetic map of tomato based on a *S. lycopersicum* × *S. pimpinellifolium* recombinant inbred line (RIL) population using 144,520 SNP markers (Gonda et al., 2019). During the past few decades, linkage maps have been utilized to decipher genetic bases of many complex traits in tomato, using various QTL mapping approaches. For example, selective genotyping, also known as trait-based analysis (TBA), identifies QTLs from selected individuals at the extreme ends of a phenotypic response distribution in a segregating population (Lebowitz et al., 1987; Foolad and Jones, 1993). This method is most useful when a single trait is being investigated, only part of the population survives the phenotyping process, a large mapping population is desired, and/or genotyping cost is a limiting factor (Lebowitz et al., 1987; Foolad and Jones, 1993; Foolad et al., 1997). Additionally, the mapping power in TBA is amplified by increasing the population size and genotyping only selected individuals with extreme phenotypes (Lumpkin et al., 2010). Previous studies have shown that when using large populations, selective genotyping of only 10% of the mapping population was sufficient to detect all major QTLs (Ayoub and Mather, 2002). In the present study, where LB resistance was the only trait being explored, a large mapping population was desired, and genotyping costs were high, QTL mapping using a TBA approach was desirable. The objectives of this study were to discover new polymorphic genetic markers (SNPs), construct a genetic linkage map, and identify QTLs responsible for LB resistance in the wild tomato species *S. pimpinellifolium* accession PI 270442. Identification of LB-resistance QTLs in PI 270442 will provide breeders with the tools to incorporate resistance from this wild accession into the cultivated tomato through marker-assisted selection (MAS). Further, the identified QTL-linked SNP markers can be used for fine mapping and ultimately identification and cloning of the underlying genes controlling LB resistance in PI 270442.

## 2 Materials and methods

### 2.1 Plant material

The LB-resistant accession PI 270442 (*S. pimpinellifolium*) was hybridized (as staminate parent) with the LB-susceptible breeding line Fla. 8059 (*S. lycopersicum*), and F_1_ seed produced in spring 2011. PI 270442 has indeterminate growth habit and produces small yellow fruit. Fla. 8059, the parent of a commercial hybrid tomato cultivar (Scott et al., 2008), has determinate growth habit and produces large red fruit. Original seed of PI 270442 was obtained from the USDA, ARS Plant Genetic Resources Unit (PGRU), Geneva, NY, USA, and that of Fla. 8059 was obtained from J.W. Scott, University of Florida, Gulf Coast Research and Education Center, Wimauma, FL, USA. A single F_1_ plant was grown in a greenhouse (GH) and self-pollinated to produce F_2_ seed during summer 2011. A large F_2_ population (n = 1,175 individuals) was grown under standard GH conditions, inoculated with *P. infestans*, and screened for response to LB disease (described below). The most resistant and most susceptible F_2_ individuals were identified and grown to maturity to produce F_3_ seed for progeny testing and confirmation of parental F_2_ phenotypes (described below). Genetic map construction and QTL mapping were performed based on the final selected resistant and susceptible F_2_ plants (n = 89; described below). Control genotypes, including LB-susceptible cultivar New Yorker (containing *Ph-1*) and LB-resistant breeding lines NC 63EB (*Ph-2*), NC 870 (*Ph-3*), and NC 03220 (*Ph-2* and *Ph-3*), and both parental lines were included in all F_2_ and F_3_ disease screening experiments. Seed of all control genotypes were initially obtained from R.G. Gardner, North Carolina State University, Mills River, NC, USA.

### 2.2 Inoculum preparation

All LB inoculations were performed using local isolates of *P. infestans*, collected from the Penn State Russell E. Larson Agricultural Research Center at Rock Springs (RS), Center County, PA. Isolate PDA-8030275 (a.k.a. RS-2004), of clonal lineage US-13, race T_1_ and mating type A-2, was used in F_2_ experiments I and II, and three of the F_3_ screening experiments; isolate RS-2009T1, of clonal lineage US-23, race T_1_ and mating type A-1, was used in F_2_ experiments III and IV, and eight of the F_3_ experiments. This difference reflected the predominant local (Rock Springs) isolates of *P. infestans* at the time of experiments (Goodwin et al., 1998; Danies et al., 2013; Gugino, 2015). All control genotypes responded the same to the two *P. infestans* isolates, allowing results to be compared across all F_2_ and F_3_ experiments.

For maintaining and propagating the pathogen isolates in an incubator, spores were collected weekly from infected leaflets and transferred to detached leaflets of a susceptible genotype. For disease screening experiments, the inoculum was prepared as previously described (Sullenberger and Foolad, 2018). Briefly, before inoculation, the sporulating leaflets were placed in a beaker containing 4°C distilled water, incubated for one hour at 4 °C, and filtered through cheesecloth to collect sporangia. The concentration of the suspension was estimated using a hemacytometer under a light microscope and adjusted to 5,000–10,000 sporangia/ml. Varying concentrations of inoculum were used across experiments to compensate for environmental conditions affecting disease pressure.

### 2.3 Plant screening for disease response and identification of F_2_ individuals with extreme phenotypes

In total, 1,175 F_2_ plants were initially screened for response to LB disease across 4 experiments during 2012 (Experiments I and II) and 2015 (Experiments III and IV). In each experiment, the two parental lines as well as three resistant (NC 63EB, NC 870, and NC 03220) and one susceptible (New Yorker) control genotypes (n = 6–24 plants/genotype) were also included. F_1_ plants (n = 24/experiment) were included in F_2_ experiments III and IV. Procedures for plant growth, inoculation, and evaluation were similar to those described in Sullenberger et al., 2018. Briefly, plants were grown in a GH in 72-cell seedling flats in a completely randomized design, using standard tomato-growing conditions. Several days before inoculation, 1–2 leaflets were collected from each F_2_ plant and stored at -80°C for later DNA extraction and genotyping. Six-week-old tomato seedlings were moved (for the remainder of the experiment) to a controlled-atmosphere GH with cooler temperatures (16–18°C), higher relative humidity (> 95% RH), and better shade control to promote LB disease infection and development. One day after moving, plants were sprayed with inoculum at a rate of 1 L per 1,000 plants twice, with a 30-minute interval between the two inoculations. Plants were scored for percent disease severity (%DS) when susceptible control genotypes showed on average > 80% DS, typically around 7–10 days after inoculation. If symptoms had not progressed significantly within 10 days of inoculation, all plants were re-inoculated. Disease severity was evaluated from 0–100%, where 0% indicated no LB disease symptom and 100% indicated complete leaf necrosis or defoliation. Tukey’s HSD test was used to compare disease responses of parental lines, F_1,_ and control genotypes.

F_2_ plants showing high levels of resistance (generally %DS ≤ 15%) or high levels of susceptibility to LB (generally %DS > 50%) were selected for mapping. Most selected plants (n = 49) were treated with the fungicide Bravo^®^ (Syngenta Crop Protection LLC, Greensboro, NC) and grown to maturity, from which F_3_ seeds were collected and grown for progeny disease testing (to confirm parental F_2_ phenotypes) between spring 2013 and spring 2014. The remaining susceptible and resistant F_2_ individuals (n = 40) did not undergo F_3_ progeny testing because of our high confidence in phenotyping during experiments III and IV. In most F_3_ progeny testing experiments, 12–18 plants per family were included in a completely randomized design. Resistant F_3_ families were those that averaged ≤ 50% DS, and susceptible F_3_ families were those with %DS > 50%. Based on the four F_2_ and 11 F_3_ screening experiments, 51 LB-resistant and 38 LB-susceptible F_2_ individuals (n = 89 extreme individual phenotypes from a total of 1,175 F_2_ plants) were selected for SNP genotyping, genetic map construction and QTL mapping, as described below.

### 2.4 Marker discovery and development

Parental lines (Fla. 8059 and PI 270442) genomic DNA was extracted using the DNeasy Plant Mini Kit (Qiagen, Seoul, Korea) and sent to BGI@UCDavis (Sacramento, CA, USA) for construction of a reduced representation library (RRL). To identify SNP markers polymorphic between the two parents, a genotyping-by-sequencing (GBS) approach was employed as previously described by Elshire et al. (2011), with some modifications. Briefly, genomic DNA was digested with NlaIII restriction enzyme and purified using AMPure PB beads (Beckman Coulter, Inc.); barcoded common adapters were ligated to the digested DNA; and samples were pooled and PCR-amplified, using AccuPrime Taq DNA polymerase. Quality was verified by measuring fragment sizes on a 2100 Bioanalyzer (Agilent Technologies, Palo Alto, CA). Most fragments were between ~170–350 bp, and adapter dimers were < 0.5%. The RRL libraries were sequenced by paired-end sequencing using Illumina HiSeq 2000 at BGI@UCDavis.

Demultiplexed FASTQ files were created using the sequencer software. CLC Genomics Workbench 8.0 (http://digitalinsights.qiagen.com) was employed to trim and clean the reads, remove any adapter remnants, and map the reads onto the tomato reference genome sequence SL2.5 (SGN, 2019). Identity between the reads and the reference map was set at 95%. A total of 19,839 SNPs were discovered using SAMtools and in-house Perl scripts (Hulse-Kemp et al., 2015) under the following requirements: a minimum read depth of 3 for a homozygous allele in both parents, polymorphism between Fla. 8059 and PI 270442, and no other SNPs within 50 bases upstream or downstream of a selected SNP.

The genetic positions of the SNPs were estimated by comparing their physical locations with markers previously mapped by the Tomato Genome Consortium (Consortium, 2012). Across the 12 tomato chromosomes, a total of 243 SNPs were selected with estimated gaps of ≤ 20 cM between them. The selected SNPs were used to develop markers, which were tested against the two parental lines using fluorescence-based Kompetitive Allele-Specific PCR (KASP) genotyping (Ag Biotech, Inc., Monterey, CA, USA). After removing markers that did not appear polymorphic or were otherwise unreliable, 212 SNP markers remained for genotyping and QTL mapping.

### 2.5 Genotyping of the selected F_2_ population

Genomic DNA of each of the 89 selected F_2_ plants (the mapping population) was extracted using a high-throughput quick DNA extraction protocol (King et al., 2014). A NanoDrop 2000 Spectrophotometer (Thermo Fisher Scientific, Waltham, MA) was used to measure the DNA concentration of each extraction, which was then adjusted to 30–60 ng/µl. KASP genotyping was performed by Ag Biotech, Inc. (https://agbiotech.net/) on the 89 selected F_2_ plants using the 212 SNP markers.

### 2.6 Linkage map construction

A genetic linkage map with 212 SNP markers was constructed using the MapMaker program v3.0b and Haldane’s mapping function (Lander et al., 1987). Markers were assigned to linkage groups (corresponding to chromosomes) using the GROUP command based on a logarithm of odds (LOD) score of 3.0. Individual linkage groups were called using the SEQUENCE command, followed by the MAP command to calculate and display a maximum likelihood map (in cM) for that linkage group. Maps were visualized by loading map distance data into the MapChart v2.3 program (Voorrips, 2002).

### 2.7 Marker segregation and trait-based QTL analysis

Marker segregation in the F_2_ population was investigated by performing a chi-square (χ^2^) goodness-of-fit test. Markers associated with QTLs for LB resistance were identified by implementing a bidirectional TBA (aka selective genotyping) approach in the F_2_ population. Allele frequency was determined for each of the 212 SNP markers in both phenotypic classes. Standard error (σ_p_) of marker allele frequency difference was calculated using (Equation 1):


(1) $${\text{σ}}_{\text{p}}={\left(\frac{p_{R}q_{R}}{2n_{R}}+\frac{p_{s}q_{s}}{2n_{S}}\right)}^{1/2}$$

where *p_R_* and *p_S_* are the PI 270442 marker allele frequencies in the resistant and susceptible classes, respectively, *q_R_* and *q_S_* are the Fla. 8059 marker allele frequencies in the resistant and susceptible classes, respectively, and *n_R_* and *n_S_* are the numbers of individuals in the F_2_ resistant and susceptible classes, respectively. Major QTLs (i.e., those with large effects; *p* < 0.01) were the ones identified with allele frequency differences *d* (*p_R_* - *p_S_*) ≥ 3σ_p_, whereas minor QTLs (i.e., those with small effects; *p* < 0.05) were the ones identified with *d* ≥ 2σ_p_ but < 3σ_p_ (Steel and Torrie, 1980; Lebowitz et al., 1987).

### 2.8 Candidate gene search

A current list of gene annotations was downloaded from ITAG4.0, which included 34,075 gene models based on tomato genome assembly SL4.0. The boundaries for each QTL were defined by the non-significant (*d* < 2σ_p_) SNP markers immediately flanking a QTL at both ends, or the end of the chromosome when the chromosome arm’s distal marker was significant. Physical locations of the SNP markers were based on SL4.0 for accurate alignment with ITAG4.0 annotations. From this subset, candidate genes were identified by manually searching gene descriptions for disease-related proteins, including major disease-resistance gene families. Candidate genes were also explored through a literature review of disease-resistance genes in tomato.

## 3 Results

### 3.1 Disease performance of the different genotypes

**Figure 1:**
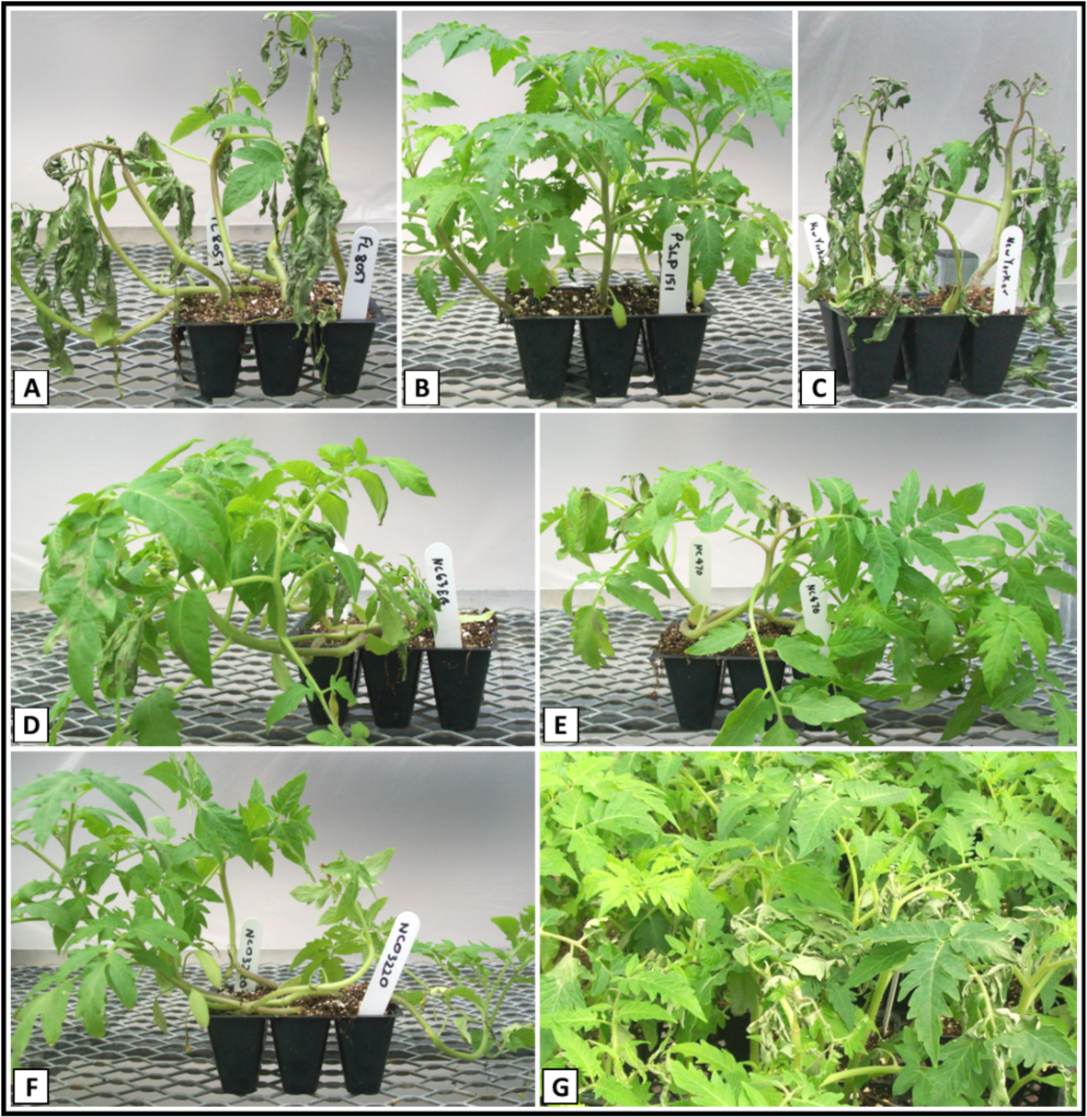
Disease response of parental, control and F_2_ plants to inoculation with *Phytophthora infestans*. **(A)** Fla. 8059; **(B)** PI 270442; **(C)** New Yorker; **(D)** NC 63EB; **(E)** NC 870; **(F)** NC 03220; **(G)** Fla. 8059 x PI 270442 F_2_.

The LB-resistant parent PI 270442 (P_2_) exhibited high levels of resistance across all experiments (avg. %DS = 4.1%), similar to the resistant control genotype NC 03220 with *Ph-2* and *Ph-3* combined resistance (**Table 1**, **Figure 1**). Across all experiments, the LB-susceptible parent Fla. 8059 (P_1_) consistently showed high levels of LB-susceptibility (avg. %DS = 89.5%), similar to the susceptible control genotype New Yorker (avg. %DS = 86.3%) (**Figure 1**). Across all experiments, the F_1_ progeny showed strong resistance (avg. %DS = 8.8%), significantly better than the resistant control lines NC 63EB (*Ph-2*; avg. %DS = 17.4%) and NC 870 (*Ph-3*; avg. %DS = 15.5%) but less than the resistant parent PI 270442 (**Table 1**). Disease responses of the parental lines, F_1_ generation and control genotypes in the current study corresponded well with our previous studies in which various isolates of *P. infestans* were used (Foolad et al., 2014; Sullenberger and Foolad, 2018).

**Table 1:** Responses of tomato plants to late blight disease in the parental and F_1_ generation and control genotypes, measured as percentage disease severity (%DS ± SE).

Genotype	Number of experiments (*n*)	Mean %DS^†^	Range
P_1_ (Fla. 8059)	15	89.5 ± 2.4	73.3-99.8
P_2_ (PI 270442)	15	4.1±0.7	0.2-10.0
F_1_	3	8.8±1.2	6.8-10.9
New Yorker (*Ph-1*)	12	86.3±3.5	68.3-100
NC 63EB (*Ph-2*)	12	17.4±3.6	2.5-51.3
NC 870 (*Ph-3*)	12	15.3±3.2	5.3-44.2
NC 03220 (*Ph-2* + *Ph-3*)	7	2.3±0.6	0-5.3

^†^ Mean comparisons were determined by Tukey’s HSD test.

**Figure 2:**
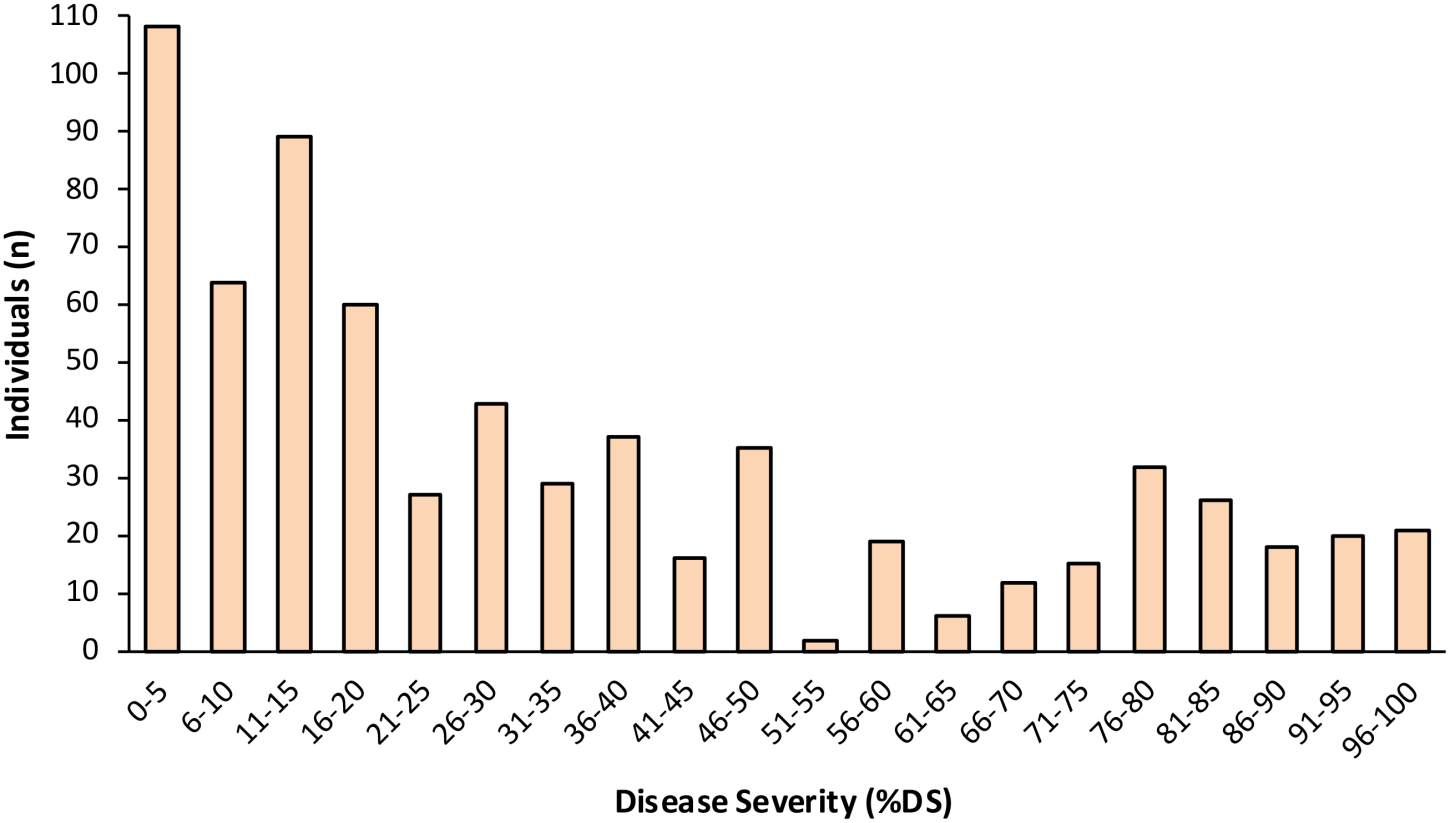
Frequency distribution of the percentage disease severity (%DS) in the F_2_ population (n = 679; from Experiments I, III and IV) derived from a cross between LB-susceptible *S. lycopersicum* breeding line Fla. 8059 and LB-resistant *S. pimpinellifolium* accession PI 270442.

In the F_2_ population (n = 1,175), %DS ranged from 0 to 100% across the 4 experiments, with the average %DS ranging from 32.7% (Exp. IV) to 46.3% (Exp. III) (**Table 2**, **Figure 1**). In F_2_ Exp. II, we had to rescue a number of highly susceptible individuals early into the experiment (i.e., before the final evaluation date) in order to prevent their death, allow recovery, grow to maturity, and collect F_3_ seed for progeny testing. Therefore, unlike in other F_2_ experiments, in F_2_ Exp. II we were not able to determine a definitive average %DS (shown as *nd* in **Table 2**). As a result of F_3_ progeny testing, six F_2_ individuals were reclassified as susceptible. The combined average %DS across experiments I, III and IV (n = 679) was 35.7%, and the combined disease distribution appeared bimodal and skewed toward resistance (**Figure 2**). Based on the F_2_ individuals’ disease performances as well as F_3_ progeny testing for disease performance, a total of 89 F_2_ individuals, including 51 most resistant (with avg. %DS = 3.9%) and 38 most susceptible (with avg. %DS = 67.6%), were selected from across the four F_2_ experiments and used for genetic mapping and QTL analysis (**Table 2**).

**Table 2:** Disease response of the F_2_ generation to infection by *P. infestans*, measured as percentage disease severity (%DS ± SD).

Genotype	Number of individuals	Mean %DS†
F_2_ Experiment I	414	33.5±28.8
F_2_ Experiment II	496	*nd*
F_2_ Experiment III	124	46.3±34.7
F_2_ Experiment IV	141	32.7±28.6
F_2_ selected individuals (resistant class)	51	3.9±2.3
F_2_ selected individuals (susceptible class)†	38	67.6±47.0

† The mean %DS for the susceptible class is based on only ten F_2_ individuals. The remaining 28 susceptible F_2_ individuals were rescued early from Experiment II for advancement to F_3_ progeny testing prior to final evaluation of the population, preventing inclusion of those plants in the selected susceptible F_2_ mean calculation; thus the mean for Experiment II was not determined (*nd*). Based on previous parent:offspring (F_2_:F_3_) correlation analysis of LB resistance in PI 270442, the actual mean %DS value of the selected susceptible class was likely much higher.

### 3.2 Marker genotyping and linkage map construction

**Figure 3:**
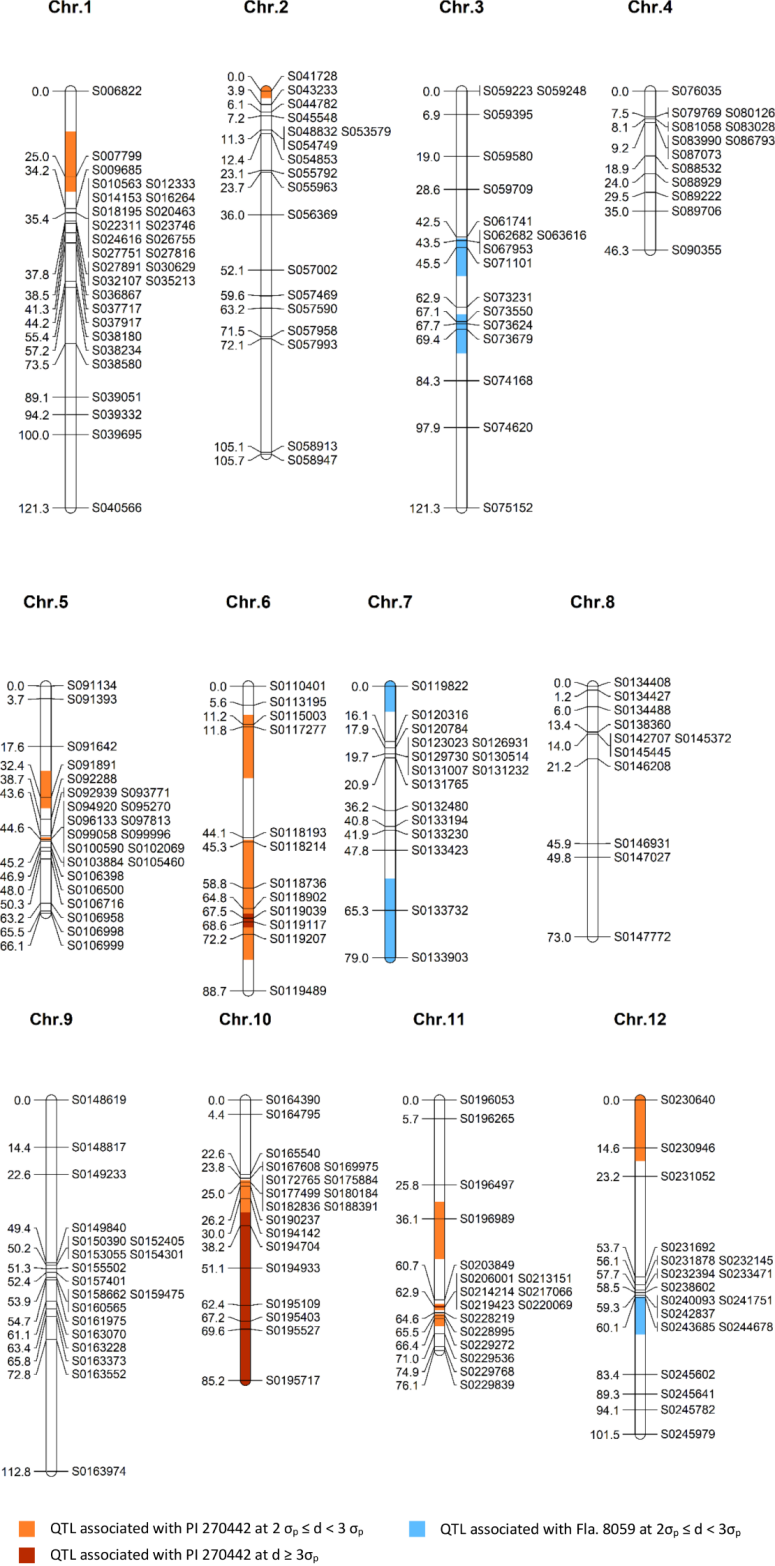
A genetic linkage map of the F_2_ population (n = 89; 212 SNP markers) derived from a cross between *S. lycopersicum* breeding line Fla. 8059 and *S. pimpinellifolium* accession PI 270442. The identified QTLs are highlighted; σ_p_ = standard error and *d* = allele frequency difference.

Genotyping of the parental lines Fla. 8059 and PI 270442 revealed 19,839 SNPs distributed across the 12 tomato chromosomes. The total SNPs per chromosome, SNP density (SNPs/Mb), and total physical distance (Mb) per chromosome are shown in **Table 3**. Initially, only 49 of the 89 selected F_2_ individuals were genotyped with 140 SNP markers with relatively even physical distribution. Genetic mapping, however, identified a few regions with “large” inter-marker gaps. This led to a second round of genotyping of all 89 selected F_2_ plants using a total of 117 SNP markers, including 69 new markers and 48 of the original 140 markers; this genotyping resulted in the elimination of “large” inter-marker gaps. The genetic linkage map was derived from all 89 F_2_ individuals and 212 SNP markers; this resulted in only 13 inter-marker gaps larger than 20 cM, which were located on chromosomes 1, 2, 3, 6, 8, 9, 11 and 12 (**Figure 3**). The chromosome length, number of markers, average physical distance, and average genetic distance between markers are shown in **Table 4**. The estimated total length of the genetic map was 1,077 cM, with each chromosome genetic length ranging from 46.3 cM (chr. 4) to 121.3 cM (chr. 1 and chr. 3) with an average of 89.8 cM (**Table 4**). The average marker density across the 12 tomato chromosomes was 4.1 cM, with 11 – 29 markers per chromosome (**Table 4**).

**Table 3:** SNPs identified between *S. lycopersicum* Fla. 8059 and *S. pimpinellifolium* PI 270442; physical distances are based on the tomato reference genome v. SL2.5.

Chromosome	# of SNPs/chr	Density (SNPs/Mb)	Total physical distance (Mb/chr)
1	1372	14	98.3
2	1188	22	54.6
3	602	9	70.5
4	1409	21	66.4
5	1853	28	65.9
6	672	14	49.5
7	556	8	68.0
8	1443	22	65.5
9	1305	18	72.3
10	931	14	65.5
11	7397	131	56.3
12	1111	17	67.0
**Total**	**19839**	**26**	**799.7**

**Table 4:** Characteristics of the linkage map constructed based on an F_2_ mapping population derived from *S. lycopersicum* (Fla. 8059) x *S. pimpinellifolium* PI 270442 cross, using 212 SNP markers; physical distances are based on the tomato reference genome v. SL2.5.

Chromosome	Chromosome length (cM)	Number of markers	Average physical distance between markers (Mb)	Average genetic distance between markers (cM)
1	121.3	29	3.8	4.3
2	105.7	18	3.1	8.2
3	121.3	17	4.3	7.5
4	46.3	13	5.0	4.2
5	66.1	23	2.9	2.9
6	88.7	12	3.2	8.1
7	79.0	16	4.4	5.3
8	73.0	11	6.3	7.3
9	112.8	19	3.8	6.3
10	85.2	19	3.6	4.7
11	76.1	17	3.2	4.8
12	101.5	18	6.1	6.0
**Total**	**1077**	**212**	**4.1**	**5.8**

### 3.3 Marker segregation

Among the 212 SNP markers, 44 (21%) exhibited segregation deviation from the expected Mendelian ratio of 1:2:1 and were located on all chromosomes but chromosome 8. Among the 44 markers, 32 showed skewed segregation in the resistant class and 19 in the susceptible class, of which 7 showed skewed segregation in both classes (**Supplementary Table 1**). It should be noted that 30 of the 44 markers with skewed segregation were at or near QTL regions associated with LB resistance (described below), and 8 markers were at the ends of chromosomes.

### 3.4 Trait-based marker analysis to identify QTLs

A comparison of marker allele frequencies (MAF) between the resistant and susceptible classes determined significant MAF differences for 46 (out of 212) markers, located in 15 genomic regions on 9 chromosomes (**Table 5**, **Figure 3**), indicating the presence of putative LB-resistance QTLs in these regions. Ten genomic regions (QTLs) were contributed from the LB-resistant parent PI 270442, and 5 from the LB-susceptible parent Fla. 8059. The ten QTLs from PI 270442 were located on chromosomes 1 (one marker), 2 (1 marker), 5 (2 locations, each including one marker), 6 (2 locations, including 2 and 6 markers), 10 (14 markers), 11 (2 locations, including 1 and 7 markers), and 12 (2 markers) (**Table 5**, **Figure 3**). The strongest effects were contributed from the QTL on chromosome 10, where 6 adjacent markers spanning 47 cM had allele frequency differences (*d*) greater than 3σ_p_, ranging from *d* = 0.25 (marker S0194704, *d* > 3σ_p_) to *d* = 0.94 (marker S0195717, *d* > 23σ_p_); for marker S0195717 within this region, 88% of the individuals in the resistant class were homozygous (*pp*) for the resistant allele (*p*) and 12% were heterozygous (*pq*), with none homozygous (*qq*) for the susceptible allele (**Table 5**). This indicated strong association of this region with LB resistance in PI 270442.

**Table 5:** Segregation of SNP markers associated with LB resistance in the F_2_ mapping population derived from *S. lycopersicum* (Fla. 8059) x *S. pimpinellifolium* PI 270442 cross; *p* and *q* indicate PI 270442 and Fla. 8059 alleles, respectively; physical distances are based on the tomato reference genome v. SL2.5.

Marker	Chromosome	Physical Locus (Mb)	Genetic Locus (cM)	Resistant Class (n = 51)					Susceptible Class (n = 38)					*p_R_-p_S_* ^b^	σ*_p_* ^c^
				*pp*	*pq*	*qq*	*p_R_* ^a^	*χ^2^* (1:2:1)	*pp*	*pq*	*qq*	*p_S_* ^a^	*χ^2^* (1:2:1)		
S007799	1	4.3	25.0	16	21	11	0.55	1.79	5	19	13	0.39	3.49	0.16*	0.08
S041728	2	0.5	0.0	14	20	14	0.50	1.33	4	12	18	0.29	14.47†	0.21*	0.08
S062682	3	25.0	43.5	1	7	8	0.28	6.38†	8	14	8	0.50	0.13	-0.22*	0.10
S067953	3	40.9	43.5	1	8	8	0.29	5.82	8	16	8	0.50	0.00	-0.21*	0.10
S071101	3	53.3	45.5	1	7	9	0.26	8.06†	7	17	8	0.48	0.19	-0.22*	0.10
S073550	3	62.4	67.1	3	29	17	0.36	9.65†	10	15	9	0.51	0.53	-0.16*	0.08
S073624	3	62.9	67.7	3	29	18	0.35	10.28†	10	17	9	0.51	0.17	-0.16*	0.08
S073679	3	63.0	69.4	3	29	18	0.35	10.28†	9	20	9	0.50	0.11	-0.15*	0.07
S091891	5	4.9	32.4	16	27	6	0.60	4.59	10	12	14	0.44	4.89	0.16*	0.08
S0102069	5	47.1	44.6	6	6	2	0.64	2.57	7	11	12	0.42	3.80	0.23*	0.11
S0115003	6	25.5	11.2	13	28	9	0.54	1.36	3	22	13	0.37	6.21†	0.17*	0.07
S0117277	6	32.8	11.8	13	28	9	0.54	1.36	3	21	14	0.36	6.79†	0.18*	0.07
S0118214	6	38.8	45.3	18	23	10	0.58	3.00	7	17	13	0.42	2.19	0.16*	0.08
S0118736	6	41.6	58.8	16	26	6	0.60	4.50	8	14	13	0.43	2.83	0.18*	0.08
S0118902	6	42.8	64.8	15	30	5	0.60	6.00†	7	15	14	0.40	3.72	0.20*	0.08
S0119039	6	43.6	67.5	15	32	4	0.61	8.06†	7	15	16	0.38	5.95	0.23**	0.07
S0119117	6	44.2	68.6	17	30	4	0.63	8.22†	7	15	16	0.38	5.95	0.25**	0.07
S0119207	6	44.8	72.2	14	33	4	0.60	8.33†	9	14	15	0.42	4.53	0.18*	0.07
S0119822	7	0.9	0.0	5	27	19	0.36	7.86†	11	15	9	0.53	0.94	-0.17*	0.08
S0133732	7	65.3	65.3	1	7	9	0.26	8.06†	9	10	10	0.48	2.86	-0.22*	0.10
S0133903	7	66.6	79.0	2	5	9	0.28	8.38†	7	16	7	0.50	0.13	-0.22*	0.10
S0172765	10	17.1	25.0	6	8	2	0.63	2.00	4	16	10	0.40	2.53	0.23*	0.11
S0175884	10	21.6	25.0	6	9	2	0.62	1.94	4	16	10	0.40	2.53	0.22*	0.10
S0177499	10	25.2	25.0	6	5	2	0.65	3.15	4	12	10	0.38	2.92	0.27*	0.12
S0180184	10	29.7	25.0	6	9	2	0.62	1.94	4	16	10	0.40	2.53	0.22*	0.10
S0182836	10	33.7	25.0	5	9	2	0.59	1.38	4	16	12	0.38	4.00	0.22*	0.11
S0188391	10	42.4	25.0	6	8	2	0.63	2.00	4	16	11	0.39	3.19	0.24*	0.11
S0190237	10	46.5	26.2	6	9	2	0.62	1.94	4	17	10	0.40	2.61	0.21*	0.10
S0194142	10	57.4	30.0	7	8	2	0.65	3.00	4	17	10	0.40	2.61	0.24*	0.10
S0194704	10	60.4	38.2	18	25	5	0.64	7.13†	2	23	10	0.39	7.11†	0.25**	0.08
S0194933	10	61.9	51.1	23	26	2	0.71	17.31†	0	24	13	0.32	12.41†	0.38**	0.07
S0195109	10	63.0	62.4	33	16	2	0.80	44.76†	0	18	20	0.24	21.16†	0.57**	0.06
S0195403	10	63.7	67.2	38	11	2	0.85	67.31†	0	17	21	0.22	23.63†	0.63**	0.06
S0195527	10	63.9	69.6	40	9	1	0.89	81.32†	0	15	23	0.20	29.53†	0.69**	0.06
S0195717	10	64.4	85.2	15	2	0	0.94	36.41†	0	0	31	0.00	93.00†	0.94**	0.04
S0196989	11	4.6	36.1	9	24	13	0.46	0.78	5	9	21	0.27	22.89†	0.19*	0.07
S0213151	11	29.9	62.9	5	8	2	0.60	1.27	4	14	12	0.37	4.40	0.23*	0.11
S0214214	11	32.1	62.9	4	9	2	0.57	1.13	3	14	12	0.34	5.62	0.22*	0.11
S0217066	11	36.2	62.9	6	8	1	0.67	3.40	3	14	12	0.34	5.62	0.32**	0.11
S0219423	11	39.2	62.9	6	6	1	0.69	3.92	4	12	12	0.36	5.14	0.34**	0.11
S0220069	11	39.9	62.9	5	7	1	0.65	2.54	3	14	12	0.34	5.62	0.31*	0.11
S0228219	11	48.9	64.6	6	7	4	0.56	1.00	4	14	13	0.35	5.52	0.20	0.10
S0228995	11	50.2	65.5	7	7	4	0.58	1.89	3	16	13	0.34	6.25†	0.24*	0.10
S0229272	11	50.6	66.4	13	18	12	0.51	1.19	5	8	15	0.32	12.29†	0.19*	0.08
S0230640	12	0.2	0.0	15	29	5	0.60	5.73	6	16	13	0.40	3.06	0.20*	0.08
S0230946	12	1.3	14.6	15	27	4	0.62	6.65†	5	20	12	0.41	2.89	0.21*	0.08
S0243685	12	55.4	60.1	7	24	18	0.39	4.96	10	20	7	0.54	0.73	-0.15*	0.08

a Frequency of PI 270442 allele in resistant (*p_R_*) and susceptible (*p_S_*) class individuals.

b Allele frequency difference between resistant and susceptible classes.

c Standard error (σ) of allele frequency difference.

† Significant deviation from 1:2:1 segregation ratio at p < 0.05.

* Allele frequency difference ≥ 2σ*_p_* (QTLs significant at p < 0.05).

** Allele frequency difference ≥ 3σ*_p_* (QTLs significant at p < 0.01).

The 2^nd^ largest-effect QTL contributed from PI 270442 was on chromosome 6, which consisted of 6 consecutive markers between 45.3 and 72.2 cM; for two of the markers, the MAF differences were greater than 3σ_p_ (**Table 5**). Also on chromosome 6, another putative QTL (the 3^rd^ QTL) was identified at the distal end of the short arm of the chromosome (11.2 – 11.8 cM) with small effects (*d* > 2σ_p_ but < 3σ_p_) (**Table 5**). The 4^th^ QTL region was identified on chromosome 11 near the centromere (62.9 – 66.4 cM), where 2 of 7 significant markers had *d* > 3σ_p_; this QTL spanned only 3.5 cM and included a marker in the middle with *d* just below 2σ_p_. The 5^th^ QTL was located on the short arm of chromosome 11, encompassing only a single marker (*d* > 2σ_p_) at 36.1 cM (**Table 5**). The 6^th^ QTL region was identified at the distal end of the short arm of chromosome 1, encompassing only one marker (*d* > 2σ_p_) at 25.0 cM. The 7^th^ QTL, also encompassing only a single marker (*d* > 2σ_p_), was located at the distal end of the short arm of chromosome 2. Two additional QTLs, 8^th^ and 9^th^, were located on chromosome 5, each encompassing only a single marker at positions 32.4 cM (short arm) and 44.6 cM (long arm), respectively. The 10^th^ LB-resistance QTL associated with PI 270442 was located on the distal end of the short arm of chromosome 12 and included 2 markers (*d* > 2σ_p_) at location 0 – 14.6 cM.

In addition to the 10 QTLs contributed from PI 270442, there were 5 putative QTLs contributed from Fla. 8059. Two were located on the long arm of chromosome 3 (three markers from 43.5 – 45.5 cM and three markers from 67.1 – 69.4 cM), two on chromosome 7 (one located at the distal end of the short arm with one marker, and one at the distal end of the long arm with two markers), and one on the long arm of chromosome 12 (with one marker); all putative QTLs associated with Fla. 8059 had generally small effects (2σ_p_ < *d* < 3σ_p_) (**Table 5**).

### 3.5 Candidate genes within QTLs

Gene models from iTAG4.0 indicated the presence of at least one candidate gene within each of 9 of the 10 QTL regions associated with LB resistance in PI 270442 (**Supplementary Table 2**). The QTL on chromosome 1, which encompassed 498 genes, included one candidate gene for a disease resistance protein. The QTL on chromosome 2 contained 24 genes, none of which was identified as being associated with disease resistance. Two QTLs on chromosome 5, mapped to the short and long arms of the chromosome, encompassed 374 and 80 genes, respectively; while the QTL on the short arm contained 11 disease resistance genes, including 3 for LB resistance, the QTL on the long arm included no resistance genes. For the two QTLs identified on the long arm of chromosome 6, the first QTL encompassed 743 genes including 2 for disease resistance, and the second QTL, with major effects (*d* > 3σ_p_), spanned 1,184 genes that included 16 disease-resistance related genes. The largest and strongest QTL, located on chromosome 10, encompassed 1,618 genes, including 25 genes for disease resistance (one for LB) or resistance gene families. The two QTLs located on the short and long arms of chromosome 11 encompassed 620 genes (including 7 for disease resistance or resistance gene families) and 607 genes (including 18 for disease resistance or resistance gene families), respectively. The last QTL, located on chromosome 12, encompassed 218 genes with 5 for disease resistance or resistance gene families. It should be noted that 5 of the 10 QTLs associated with LB resistance in PI 270442 (chromosomes 1, 2, 6, 10, and 11) corresponded with the previously-known LB-resistance genes or QTLs in tomato and 5 were new (see Discussion).

## 4 Discussion

The parental breeding line Fla. 8059 showed high LB susceptibility across all experiments, and the *S. pimpinellifolium* parental line PI 270442 exhibited strong LB resistance across all experiments; the disease response of accession PI 270442 was similar to the resistant control line NC 03220 that contains *Ph-2* and *Ph-3* combined resistance genes in homozygous conditions (**Table 1**, **Figure 1**). The results highlight the potential value of PI 270442 as a genetic resource for LB resistance breeding in tomato. The F_1_ generation, derived from the cross between the two parental lines, exhibited strong LB resistance, significantly better than the resistant control lines NC 63EB (*Ph-2*) and NC 870 (*Ph-3*) but not as strong as PI 270442 or NC 03220 (*Ph-2* + *Ph-3*) (**Table 1**); this indicates the presence of some dominance effects for LB resistance in accession PI 270442, and thus its utility for developing F_1_ hybrid cultivars of tomato with LB resistance.

Across F_2_ experiments I, III and IV (n = 679), the %DS ranged from 0 – 100% (**Table 2**), averaging 35.7% with somewhat bimodal distribution skewed toward resistance (**Figure 2**). The distribution pattern indicates involvement of one or a few loci acting with dominance or partial dominance gene action, a finding also supported by our inheritance study of an F_2_ population of the same cross (Sullenberger and Foolad, 2018). The results of the current QTL study are in agreement, with the identification of 3 major resistance QTLs and with most of the variation attributed to a single QTL.

When performing selective genotyping for QTL analysis, the individuals with most extreme phenotypes in the population must be identified and confirmed unequivocally for accurate genetic mapping. In the present study, over half (49) of the selected F_2_ plants were advanced to F_3_ generation and progeny families tested for response to LB disease. For most of those, the initial phenotypes were confirmed upon F_3_ progeny testing and only 6 plants (6.7%) were discovered to have been incorrectly phenotyped; this demonstrates the importance of progeny testing and phenotype confirmation to improve the accuracy of QTL analysis.

After sequencing the two parental lines (PI 270442 and Fla. 8059), 19,839 SNPs were identified, of which 212 SNP markers, from an initial selection of 243 uniformly distributed SNPs (87%), produced informative KASP markers. The accuracy of KASP genotyping using the 212 SNP markers was estimated to be 96%, based on the number of useful data points for each marker. The genetic map positions of the 212 SNP markers were confirmed by linkage analysis in the F_2_ mapping population, which indicated a genome size of 1,077 cM (**Table 4**) that was similar to previous reports in other *S. lycopersicum* × *S. pimpinellifolium* mapping populations (Sharma et al., 2008; Ashrafi et al., 2012; Merk et al., 2012; Ohlson et al., 2018; Sullenberger et al., 2022). The map positions of the markers were also in agreement with two other recently published *S. lycopersicum* × *S. pimpinellifolium* genetic maps, which were constructed based on the same/similar SNP markers (Ohlson et al., 2018; Sullenberger et al., 2022). In the present study, the genetic map encompassed a good coverage of the tomato chromosomes, with an average inter-marker distance of 5.8 cM and with 94% of the inter-marker spaces being less than 20 cM.

Comparisons of the genetic map locations of the 212 SNP markers with their physical map locations in the tomato genome version SL2.5 revealed good correspondences; however, when compared with the tomato genome version SL4.0, the genetic positions of a few markers (in 2 genomic locations) did not match. Specifically, SNP marker S0164390, which was mapped to the distal end of the short arm of chromosome 10 in both the genetic map (**Figure 3**) and SL2.5 physical map, was mapped to the distal end of the long arm of chromosome 10 in SL4.0. Based on our QTL analyses, other markers at the distal end of the long arm of chromosome 10 showed strong association with LB resistance whereas marker S0164390 did not show such association. We conclude, therefore, that the correct position of marker S0164390 is most likely the placement shown on the genetic linkage map and the SL2.5 physical map. Additionally, 5 consecutive SNP markers (S0240093, S0241751, S0242837, S0243685 and S0244678), located on chromosome 12, are inverted in SL4.0 relative to SL2.5; all 5 markers were mapped to within 0.8 cM of each other and two are associated with LB resistance (see below).

Upon a chi-square (χ^2^) goodness-of-fit test, it was determined that 79% of the SNP markers showed normal segregation based on the expected Mendelian ratio of 1:2:1 in both the selected resistant and selected susceptible F_2_ classes. Skewed segregation, however, was observed for 44 markers (21%) in one or both classes, of which the genetic locations of 30 markers (68%) were associated with LB resistance and thus their skewness is presumed to be due to the selections applied in the F_2_ population. As to the remaining 14 skewed markers (6.6% of the total), the level of skewness was highly conformable to the previously-published *S. lycopersicum* × *S. pimpinellifolium* interspecific populations (e.g. Ashrafi et al., 2009, 2012; Foolad et al., 2015; Ohlson et al., 2018; Sullenberger et al., 2022). Because of our bidirectional selective genotyping approach, any skewed segregation due to factors other than LB response is unlikely to affect the accuracy of QTL identification (Navabi et al., 2009).

Selective genotyping, as performed in this study, has been proven to be an effective approach for QTL identification in several other tomato populations (Foolad et al., 1997; Merk et al., 2012; Ohlson et al., 2018; Sullenberger et al., 2022). According to previous studies, selective genotyping of 10% of a mapping population is sufficient to detect all major QTLs (described in Introduction). Further, selecting 6% of a mapping population of 500 individuals would identify most QTLs that have ≥ 15% effect, with the power of QTL detection increasing with the population size (Navabi et al., 2009). Here, we employed TBA and genotyped ~8% of the F_2_ individuals (the extreme ends of the disease response distribution) from a population of > 1,100 individuals, which provided strong confidence that all significant QTLs associated with LB resistance would be detected.

Of the 10 QTLs detected in PI 270442 for LB resistance, five (on chromosomes 1, 2, 6, 10 and 11) co-localized with LB-resistance QTLs previously reported in other wild tomato accessions (Merk et al., 2012; Chen et al., 2014; Arafa et al., 2017; Sullenberger et al., 2022) and five (on chromosomes 5, 6, 11 and 12) appeared to be new. The QTL on the short arm of chromosome 1 contained a single marker (S007799) that mapped to the genetic location at 25 cM (**Figure 3**) and encompassed a physical region of 4.3 Mb (**Table 5**), which is 1.5 Mb away from a gene encoding a TIR-NBS-LRR resistance protein (**Supplementary Table 2**); marker S007799 was also recently reported to be associated with LB resistance in another *S. pimpinellifiolium* accession (Sullenberger et al., 2022). The second QTL was identified at the distal end of the short arm of chromosome 2 and was associated with one marker, S041728 (**Table 5**; **Figure 3**); this marker coincides with the location of a LB-resistance QTL previously identified in *S. pimpinellifiolium* wild accession L3707 (Chen et al., 2014) (**Supplementary Table 2**). The two LB-resistance QTLs identified on chromosome 5 of PI 270442 each included only one marker (**Table 5**): the first one (associated with marker S091891), a newly identified QTL for LB resistance in tomato, was located on the short arm of the chromosome at 32.4 cM and 4.9 Mb, and there are 11 disease-resistance genes associated with this QTL, three of which encode for predicted LB-resistance proteins and one for a CC-NBS-LRR protein (**Supplementary Table 2**); the second chromosome 5 QTL, associated with marker S0102069, was mapped to the long arm at 44.6 cM and 47.1 Mb (**Table 5**, **Figure 3**), and there are no known disease-resistance or LB-resistance genes/QTLs in this location.

For the two QTLs detected on the long arm of chromosome 6 of PI 270442, the first one spanned 2 markers with inter-marker genetic distance of 0.6 cM and physical space of 7.3 Mb (**Table 5**; **Figure 3**); this region contains 2 genes for disease resistance (**Supplementary Table 2**) with no previous report of a LB-resistance gene or QTL at this location. The second QTL on chromosome 6, spanning 6 markers with 26.9 cM genetic distance and 6.0 Mb physical space (**Table 5**, **Figure 3**), contains 16 genes related to disease resistance (**Supplementary Table 2**), of which two are TIR-NBS-LRR genes, two are NB-LRR tospovirus immune receptors, and two are NBS-coding resistance gene analogs; a previous mapping study in the tomato wild species *S. habrochaites* identified a 6.8 Mb QTL for LB resistance that corresponds with this region (Arafa et al., 2017).

The strongest QTL for LB resistance in the present study was identified on chromosome 10, encompassing 14 markers, 60.2 cM genetic distance, and 47.3 Mb physical space; this locus extended from near the centromere to the distal end of the long arm of the chromosome, where the strongest effects were exerted. The allele frequency difference (*d*) between the two selected classes for the distal marker was > 23σ_p_, with 96% of the individuals in the selected resistant and selected susceptible classes were homozygous for the allele contributed by the resistant (PI 270442) and susceptible (Fla. 8059) parents, respectively. This demonstrates the significance of this QTL in providing the strong LB resistance conferred by PI 270442. This QTL region includes 25 genes for disease resistance, 4 of which encode NBS-LRR proteins and one encoding a LB-resistance protein (**Supplementary Table 2**). Previous reports placed *Ph-2* LB-resistance gene (Moreau et al., 1998; Zhi et al., 2021) and *Ph-5-2* LB-resistance QTL (Merk et al., 2012) at this location in other *S. pimpinellifolium* accessions. This chromosome 10 region also corresponds with LB resistance in several wild species of potato (Park et al., 2009; Brylińska et al., 2015). It is not known whether *Ph-2* gene or other genes within the same resistance gene family are responsible for the resistance observed across tomato and potato species. Further investigation is needed to help determine the nature of this QTL in PI 270442.

Chromosome 11 contained two LB-resistance QTLs: a single-marker QTL on the short arm of the chromosome at genetic position of 36.1 cM and physical position of 4.6 Mb (**Table 5**, **Figure 3**); this QTL is within 5.7 Mb space of 7 disease-resistance genes, four of which encoding TIR-NBS-LRR proteins (with 3 within 0.16 – 0.39 Mb distance from the significant marker) and one encoding an NBS-LRR protein (**Supplementary Table 2**). The second QTL is located on the long arm of the chromosome and includes 8 consecutive significant markers (one approaching significance) spanning 3.5 cM and 20.6 Mb (**Table 5**, **Figure 3**); this QTL contains 18 disease-resistance related genes, two encoding TIR-NBS-LRR proteins and 13 encoding CC-NBS-LRR proteins, and all of them within 0.84 Mb of at least one significant marker. Three markers within this chromosome 11 QTL region (markers S0214214, S0217066, and S0220069) were previously reported to be associated with LB resistance in a different accession of *S. pimpinellifolium* (Sullenberger et al., 2022). Further, at this location, QTLs/genes for resistance to early blight (Foolad et al., 2002) and fusarium wilt (Sarfatti et al., 1989) were previously reported. The final (and 10^th^) QTL conferring LB resistance in PI 270442, at the distal end of the short arm of chromosome 12 (**Table 5**, **Figure 3**), spans 14.6 cM genetic distance and 1.1 Mb physical region and contains 5 genes for disease resistance, two of which encode NBS-LRR proteins (**Supplementary Table 2**).

Five QTLs were also detected in the LB-susceptible parent Fla. 8059, which contributed to the better performance of the F_2_ plants under LB disease pressure. Of these, 2 QTLs were located on the long arm of chromosome 3, each including 3 markers significant only at the 5% level (*d* > 2σ_p_), 2 QTLs at the distal ends of the long and short arms of chromosome 7, each represented by one and 2 markers, respectively, and significant only at the 5% level (*d* > 2σ_p_), and one QTL on the distal end of the short arm of chromosome 12, represented by a single marker significant at the 5% level (**Table 5**). The three Fla. 8059 QTLs on chromosomes 7 and 12 were located at the distal ends of chromosomes, each represented by 1 or 2 markers, indicating that they might be false QTLs. This conclusion is supported by numerous previous studies (as well as the current study) demonstrating that Fla. 8059 is highly susceptible to LB, and that the identified QTLs might be the result of Fla. 8059’s strong vigor rather than a genetic disease resistance.

It is worth noting that PI 270442 exhibits stronger LB resistance than the *S. pimpinellifolium* accession L3708, the source of *Ph-3* and the strongest commercially available LB-resistance gene in tomato (Foolad et al., 2014; Gugino et al., 2014; Foolad et al., 2015). Resistance in PI 270442 is also stronger than the commercial breeding lines or hybrid cultivars containing *Ph-3* alone (Foolad et al., 2014). While no QTL is identified in PI 270442 in the *Ph-3* region, its LB resistance is nonetheless similar to the lines and hybrid cultivars that contain *Ph-2 + Ph-3* combined (Foolad et al., 2014), suggesting the presence of additional, most likely new, LB resistance genes in this accession. The largest QTL identified in the present study in PI 270442 co-localized with *Ph-2* on chromosome 10; however, *Ph-2* resistance gene alone, in advanced breeding lines (Foolad et al., 2014) or original wild genetic backgrounds (Goodwin et al., 1995) could only slow down disease progression and is ineffective against aggressive isolates of *P. infestans* (Moreau et al., 1998; Foolad et al., 2008). We have shown across multiple studies that PI 270442 exhibits stronger LB resistance than lines containing *Ph-2* alone (**Table 1**), indicating that the LB resistance in PI 270442 is the result of multiple genes that may work in concert with *Ph-*2 or an entirely different gene(s) within the *Ph-2* region (Foolad et al., 2014, 2015; Sullenberger and Foolad, 2018). Further, of the total of 10 LB-resistance QTLs identified in the present study in PI 270442, only 5 were previously identified in other tomato wild accessions, and only 3 of them overlap with the LB-resistance QTLs we recently identified in *S. pimpinellifolium* accession PI 270441. The overall results across various studies indicate the presence of diverse and abundant LB-resistance genes/QTLs among *S. pimpinellifolium* wild accessions, which could be utilized in tomato breeding to develop new breeding lines and hybrid cultivars with strong and durable LB resistance.

The consistent and exceptional performance of PI 270442 against LB across multiple studies demonstrates its value as a source of breeding material for LB resistance in tomato. The LB-resistance QTLs and associated markers identified in the present study can facilitate transferring resistance from PI 270442 into the cultivated tomato. While most of the QTLs identified in this study are not currently used in tomato breeding, the marker-QTL information provided here will be useful for future investigation and practical applications, including 1) marker-assisted breeding for LB resistance in tomato using the QTL-linked markers, 2) fine mapping of the identified QTLs using polymorphic markers from among the 19,839 SNPs identified between Fla. 8059 and PI 270442, 3) accurate identification of candidate genes underlying the identified LB-resistance QTLs, and 4) map-based or shotgun cloning of genes underlying the identified LB-resistance QTLs. Of particular importance is to determine the relationship between the LB-resistance QTL identified on chromosome 10 in the present study and the previously known LB-resistance gene *Ph-2*.

## 5 Conclusion

Nearly 20,000 SNPs were identified between the two parental lines of this study (PI 270442 and Fla. 8059) and a genetic linkage map with 212 SNP markers was constructed using an F_2_ population. Ten LB-resistance QTLs were identified in PI 270442 on chromosomes 1, 2, 5, 6, 10, 11 and 12, with the strongest QTLs located on chromosomes 6, 10 and 11. The identified QTL-linked markers can be employed in breeding programs to transfer LB resistance from PI 270442 into the cultivated tomato via marker-assisted breeding and to develop near-isogenic lines for fine mapping of the QTLs. A comparison of the genomic locations of the QTLs with the tomato physical map led to the identification of several candidate genes, which might be underpinning the LB resistance in PI 270442.

## Data Availability

The original contributions presented in the study are publicly available. This data can be found here: NCBI, PRJNA471200.
