# Supplementary material for: Identification and mapping of QTLs for late blight resistance in the wild tomato (Solanum pimpinellifolium) accession PI 270442 via selective genotyping
Source: Front Plant Sci. 2024 Nov 15;15:1482241. doi: 10.3389/fpls.2024.1482241 (PMC11604435; doi:10.3389/fpls.2024.1482241)
Supplement: Supplementary file 1 [file DataSheet1.pdf]

Table S1. Marker allele frequencies for the selected resistant and susceptible F_2 plants, where *p* and *q* indicate *S. pimpinellifolium* accession PI 270442 and *S. lycopersicum* breeding line Fla. 8059 alleles, respectively. Chromosome and physical locus positions are based on the tomato genome release SL2.5. The frequency of PI 270442 alleles in the resistant and susceptible classes are denoted by p_R and p_S, respectively

				Resistant Class					Susceptible Class				
Marker	Chromosome	Physical Locus (Mb)	Genetic Locus (cM)	*pp*	*pq*	*qq*	p_R	χ^2 (1:2:1)	*pp*	*pq*	*qq*	p_S	χ^2 (1:2:1)
S006822	1	0.9	0.0	17	18	14	0.53	3.82	8	19	10	0.47	0.24
S007799	1	4.3	25.0	16	21	11	0.55	1.79	5	19	13	0.39	3.49
S009685	1	10.0	34.2	6	4	5	0.53	3.40	5	13	12	0.38	3.80
S010563	1	12.0	35.4	6	6	4	0.56	1.50	5	15	12	0.39	3.19
S012333	1	15.6	35.4	6	6	4	0.56	1.50	5	15	11	0.40	2.35
S014153	1	19.2	35.4	5	4	4	0.54	2.08	5	14	12	0.39	3.45
S016264	1	23.1	35.4	6	7	4	0.56	1.00	5	15	12	0.39	3.19
S018195	1	27.1	35.4	6	7	4	0.56	1.00	5	15	12	0.39	3.19
S020463	1	31.4	35.4	6	7	4	0.56	1.00	5	15	12	0.39	3.19
S022311	1	39.8	35.4	6	7	4	0.56	1.00	5	15	12	0.39	3.19
S023746	1	43.0	35.4	6	7	4	0.56	1.00	5	14	12	0.39	3.45
S024616	1	45.2	35.4	4	4	4	0.50	1.33	4	15	10	0.40	2.52
S026755	1	48.4	35.4	6	7	4	0.56	1.00	5	15	12	0.39	3.19
S027751	1	51.5	35.4	6	6	4	0.56	1.50	4	14	11	0.38	3.41
S027816	1	52.0	35.4	6	5	4	0.57	2.20	4	13	11	0.38	3.64
S027891	1	52.5	37.8	6	4	5	0.53	3.40	5	14	13	0.38	4.50
S030629	1	56.6	37.8	6	6	3	0.60	1.80	5	14	12	0.39	3.45
S032107	1	59.5	37.8	6	5	5	0.53	2.38	5	13	11	0.40	2.79
S035213	1	67.1	37.8	15	24	12	0.53	0.53	6	17	14	0.39	3.70
S036867	1	72.7	38.5	6	4	6	0.50	4.00	5	14	13	0.38	4.50
S037717	1	76.2	41.3	15	24	12	0.53	0.53	7	16	14	0.41	3.32
S037917	1	77.4	44.2	15	24	11	0.54	0.72	6	18	13	0.41	2.68
S038180	1	79.8	55.4	15	24	11	0.54	0.72	6	20	10	0.44	1.33

S038234	1	80.1	57.2	15	25	11	0.54	0.65	8	20	9	0.49	0.30
S038580	1	82.8	73.5	10	30	8	0.52	3.17	10	19	8	0.53	0.24
S039051	1	86.0	89.1	10	29	8	0.52	2.74	12	14	9	0.54	1.91
S039332	1	87.7	94.2	10	33	8	0.52	4.57	11	16	9	0.53	0.67
S039695	1	89.7	100.0	10	32	6	0.54	6.00†	11	15	10	0.51	1.06
S040566	1	93.9	121.3	10	30	6	0.54	4.96	10	20	7	0.54	0.73
S041728	2	0.5	0.0	14	20	14	0.50	1.33	4	12	18	0.29	14.47†
S043233	2	3.4	3.9	14	13	18	0.46	8.73†	5	13	11	0.40	2.79
S044782	2	6.2	6.1	5	4	5	0.50	2.57	4	13	9	0.40	1.92
S045548	2	7.4	7.2	5	6	6	0.47	1.59	4	14	11	0.38	3.41
S048832	2	15.2	11.3	7	5	5	0.56	3.35	4	19	8	0.44	2.61
S053579	2	25.8	11.3	7	4	5	0.56	4.50	4	18	7	0.45	2.31
S054749	2	31.2	11.3	16	18	17	0.49	4.45	4	21	13	0.38	4.68
S054853	2	32.9	12.4	15	18	17	0.48	4.08	5	19	14	0.38	4.26
S055792	2	36.6	23.1	17	15	18	0.49	8.04†	7	19	11	0.45	0.89
S055963	2	37.4	23.7	17	15	18	0.49	8.04†	8	19	11	0.46	0.47
S056369	2	39.5	36.0	17	19	14	0.53	3.24	7	20	11	0.45	0.95
S057002	2	42.4	52.1	20	21	10	0.60	5.51	8	21	9	0.49	0.47
S057469	2	44.6	59.6	18	21	10	0.58	3.61	8	26	4	0.55	6.00†
S057590	2	45.5	63.2	21	20	10	0.61	7.12†	9	24	5	0.55	3.47
S057958	2	47.7	71.5	19	21	10	0.59	4.52	12	18	7	0.57	1.38
S057993	2	47.8	72.1	18	21	10	0.58	3.61	12	20	6	0.58	2.00
S058913	2	53.6	105.1	15	25	8	0.57	2.13	13	19	5	0.61	3.49
S058947	2	53.9	105.7	15	29	7	0.58	3.47	13	19	5	0.61	3.49
S059223	3	0.9	0.0	14	30	7	0.57	3.51	11	15	10	0.51	1.06
S059248	3	1.0	0.0	14	29	7	0.57	3.24	12	16	10	0.53	1.16
S059395	3	1.9	6.9	9	32	8	0.51	4.63	11	17	7	0.56	0.94
S059580	3	2.8	19.0	8	26	11	0.47	1.49	9	12	13	0.44	3.88
S059709	3	3.7	28.6	8	31	11	0.47	3.24	10	17	10	0.50	0.24

S061741	3	19.9	42.5	7	29	14	0.43	3.24	9	19	10	0.49	0.05
S062682	3	25.0	43.5	1	7	8	0.28	6.38†	8	14	8	0.50	0.13
S063616	3	28.9	43.5	1	8	8	0.29	5.82	7	16	8	0.48	0.10
S067953	3	40.9	43.5	1	8	8	0.29	5.82	8	16	8	0.50	0.00
S071101	3	53.3	45.5	1	7	9	0.26	8.06†	7	17	8	0.48	0.19
S073231	3	60.8	62.9	3	30	15	0.38	9.00†	8	20	10	0.47	0.32
S073550	3	62.4	67.1	3	29	17	0.36	9.65†	10	15	9	0.51	0.53
S073624	3	62.9	67.7	3	29	18	0.35	10.28†	10	17	9	0.51	0.17
S073679	3	63.0	69.4	3	29	18	0.35	10.28†	9	20	9	0.50	0.11
S074168	3	65.0	84.3	5	32	14	0.41	6.49†	9	23	5	0.55	3.05
S074620	3	67.0	97.9	1	7	6	0.32	3.57	6	17	7	0.48	0.60
S075152	3	70.4	121.3	3	8	5	0.44	0.50	6	20	6	0.50	2.00
S076035	4	4.5	0.0	3	8	6	0.41	1.12	4	22	2	0.54	9.43†
S079769	4	16.9	7.5	10	28	13	0.47	0.84	8	21	9	0.49	0.47
S080126	4	18.4	7.5	3	9	5	0.44	0.53	6	18	8	0.47	0.75
S081058	4	22.3	8.1	10	28	12	0.48	0.88	8	20	9	0.49	0.30
S083028	4	31.8	8.1	3	8	1	0.58	2.00	6	18	7	0.48	0.87
S083990	4	35.9	9.2	4	8	4	0.50	0.00	6	18	8	0.47	0.75
S086793	4	47.1	9.2	3	9	4	0.47	0.38	6	18	8	0.47	0.75
S087073	4	48.3	9.2	4	8	4	0.50	0.00	6	18	8	0.47	0.75
S088532	4	55.8	18.9	11	26	12	0.49	0.22	10	17	8	0.53	0.26
S088929	4	59.3	24.0	11	27	13	0.48	0.33	11	20	7	0.55	0.95
S089222	4	60.3	29.5	9	17	6	0.55	0.69	1	4	1	0.50	0.67
S089706	4	62.5	35.0	13	22	9	0.55	0.73	13	16	8	0.57	2.03
S090355	4	64.9	46.3	12	29	9	0.53	1.64	13	15	7	0.59	2.77
S091134	5	0.5	0.0	16	27	8	0.58	2.69	9	17	11	0.47	0.46
S091393	5	1.7	3.7	15	26	7	0.58	3.00	12	13	11	0.51	2.83
S091642	5	3.2	17.6	14	27	8	0.56	1.98	9	19	10	0.49	0.05
S091891	5	4.9	32.4	16	27	6	0.60	4.59	10	12	14	0.44	4.89

S092288	5	6.8	38.7	5	7	2	0.61	1.29	7	11	14	0.39	6.19†
S092939	5	10.2	43.6	6	9	2	0.62	1.94	8	13	11	0.45	1.69
S093771	5	14.1	43.6	6	9	1	0.66	3.38	8	12	10	0.47	1.47
S094920	5	18.1	44.6	16	26	7	0.59	3.49	10	15	12	0.47	1.54
S095270	5	20.4	44.6	16	25	8	0.58	2.63	10	15	13	0.46	2.16
S096133	5	24.6	44.6	6	9	2	0.62	1.94	8	12	11	0.45	2.16
S097813	5	33.2	44.6	6	9	2	0.62	1.94	8	12	11	0.45	2.16
S099058	5	36.7	44.6	5	9	2	0.59	1.38	8	10	12	0.43	4.40
S099996	5	40.1	44.6	6	9	2	0.62	1.94	8	11	12	0.44	3.65
S0100590	5	43.8	44.6	6	9	2	0.62	1.94	8	12	12	0.44	3.00
S0102069	5	47.1	44.6	6	6	2	0.64	2.57	7	11	12	0.42	3.80
S0103884	5	52.6	45.2	5	9	2	0.59	1.38	8	13	10	0.47	1.06
S0105460	5	59.1	45.2	15	27	8	0.57	2.28	9	16	12	0.46	1.16
S0106398	5	61.5	46.9	17	26	7	0.60	4.08	11	15	12	0.49	1.74
S0106500	5	61.7	48.0	16	28	7	0.59	3.67	11	14	13	0.47	2.84
S0106716	5	62.6	50.3	15	31	4	0.61	7.72†	11	14	13	0.47	2.84
S0106958	5	64.0	63.2	14	29	6	0.58	4.27	14	12	12	0.53	5.37
S0106998	5	64.2	65.5	14	31	5	0.59	6.12†	13	12	12	0.51	4.62
S0106999	5	64.2	66.1	14	29	5	0.59	5.46	11	13	13	0.47	3.49
S0110401	6	12.6	0.0	3	8	6	0.41	1.12	3	13	16	0.30	11.69†
S0113195	6	20.2	5.6	3	10	3	0.50	1.00	3	15	9	0.39	3.00
S0115003	6	25.5	11.2	13	28	9	0.54	1.36	3	22	13	0.37	6.21†
S0117277	6	32.8	11.8	13	28	9	0.54	1.36	3	21	14	0.36	6.79†
S0118193	6	38.8	44.1	17	23	10	0.57	2.28	6	19	11	0.43	1.50
S0118214	6	38.8	45.3	18	23	10	0.58	3.00	7	17	13	0.42	2.19
S0118736	6	41.6	58.8	16	26	6	0.60	4.50	8	14	13	0.43	2.83
S0118902	6	42.8	64.8	15	30	5	0.60	6.00†	7	15	14	0.40	3.72
S0119039	6	43.6	67.5	15	32	4	0.61	8.06†	7	15	16	0.38	5.95
S0119117	6	44.2	68.6	17	30	4	0.63	8.22†	7	15	16	0.38	5.95

S0119207	6	44.8	72.2	14	33	4	0.60	8.33†	9	14	15	0.42	4.53
S0119489	6	47.4	88.7	3	9	3	0.50	0.60	5	22	4	0.52	5.52
S0119822	7	0.9	0.0	5	27	19	0.36	7.86†	11	15	9	0.53	0.94
S0120316	7	4.1	16.1	5	29	16	0.39	6.12†	8	17	11	0.46	0.61
S0120784	7	7.1	17.9	5	23	14	0.39	4.24	7	13	11	0.44	1.84
S0123023	7	17.3	19.7	6	30	15	0.41	4.76	9	18	11	0.47	0.32
S0126931	7	32.9	19.7	1	9	7	0.32	4.29	6	16	10	0.44	1.00
S0129730	7	44.9	19.7	1	7	4	0.38	1.83	6	13	10	0.43	1.41
S0130514	7	48.2	19.7	0	9	7	0.28	6.38†	6	16	10	0.44	1.00
S0131007	7	50.2	19.7	0	8	7	0.27	6.60†	6	15	9	0.45	0.60
S0131232	7	51.1	19.7	1	9	7	0.32	4.29	6	16	10	0.44	1.00
S0131765	7	54.3	20.9	6	30	15	0.41	4.76	9	16	11	0.47	0.67
S0132480	7	59.7	36.2	7	25	18	0.39	4.84	9	14	15	0.42	4.53
S0133194	7	61.0	40.8	1	8	8	0.29	5.82	8	11	11	0.45	2.73
S0133230	7	61.2	41.9	7	25	17	0.40	4.10	9	14	12	0.46	1.91
S0133423	7	62.7	47.8	8	25	17	0.41	3.24	12	13	13	0.49	3.84
S0133732	7	65.3	65.3	1	7	9	0.26	8.06†	9	10	10	0.48	2.86
S0133903	7	66.6	79.0	2	5	9	0.28	8.38†	7	16	7	0.50	0.13
S0134408	8	1.7	0.0	9	30	9	0.50	3.00	6	17	14	0.39	3.70
S0134427	8	1.8	1.2	4	10	3	0.53	0.65	5	14	12	0.39	3.45
S0134488	8	2.3	6.0	2	12	3	0.47	3.00	5	16	10	0.42	1.65
S0138360	8	20.1	13.4	13	26	12	0.51	0.06	6	18	12	0.42	2.00
S0142707	8	39.0	14.0	11	26	12	0.49	0.22	6	18	13	0.41	2.68
S0145372	8	50.5	14.0	4	8	5	0.47	0.18	4	16	11	0.39	3.19
S0145445	8	50.8	14.0	12	26	12	0.50	0.08	6	17	12	0.41	2.09
S0146208	8	56.5	21.2	7	29	11	0.46	3.26	8	17	12	0.45	1.11
S0146931	8	60.6	45.9	6	15	11	0.42	1.69	2	0	3	0.40	5.40
S0147027	8	61.0	49.8	7	29	15	0.42	3.47	6	23	8	0.47	2.41
S0147772	8	65.0	73.0	8	27	15	0.43	2.28	6	22	7	0.49	2.37

S0148619	9	1.5	0.0	12	20	17	0.45	2.67	4	19	13	0.38	4.61
S0148817	9	2.1	14.4	10	21	16	0.44	2.06	5	14	12	0.39	3.45
S0149233	9	3.6	22.6	12	18	20	0.42	6.48†	4	13	16	0.32	10.21†
S0149840	9	6.4	49.4	13	24	14	0.49	0.22	8	17	12	0.45	1.11
S0150390	9	9.1	50.2	5	7	4	0.53	0.38	7	14	9	0.47	0.40
S0152405	9	17.4	50.2	6	7	4	0.56	1.00	8	14	9	0.48	0.35
S0153055	9	22.2	50.2	5	6	4	0.53	0.73	7	14	8	0.48	0.10
S0154301	9	27.2	50.2	6	7	4	0.56	1.00	8	14	9	0.48	0.35
S0155502	9	31.3	51.3	5	6	4	0.53	0.73	6	15	9	0.45	0.60
S0157401	9	39.4	52.4	5	4	6	0.47	3.40	7	14	9	0.47	0.40
S0158662	9	43.1	53.9	6	6	4	0.56	1.50	8	14	9	0.48	0.35
S0159475	9	45.5	53.9	6	6	2	0.64	2.57	8	13	9	0.48	0.60
S0160565	9	50.2	53.9	6	3	3	0.63	4.50	5	12	8	0.44	0.76
S0161975	9	57.6	54.7	6	7	4	0.56	1.00	8	14	10	0.47	0.75
S0163070	9	63.7	61.1	7	5	5	0.56	3.35	7	16	8	0.48	0.10
S0163228	9	64.8	63.4	15	22	11	0.54	1.00	7	19	7	0.50	0.76
S0163373	9	65.9	65.8	16	19	14	0.52	2.63	6	19	11	0.43	1.50
S0163552	9	67.1	72.8	14	24	12	0.52	0.24	6	20	11	0.43	1.59
S0163974	9	69.9	112.8	4	5	5	0.46	1.29	2	16	11	0.34	5.90
S0164390	10	0.0	0.0	11	28	11	0.50	0.72	6	17	13	0.40	2.83
S0164795	10	1.8	4.4	9	29	12	0.47	1.64	5	19	12	0.40	2.83
S0165540	10	4.8	22.6	13	23	12	0.51	0.13	4	18	13	0.37	4.66
S0167608	10	9.3	23.8	6	7	3	0.59	1.38	4	15	10	0.40	2.52
S0169975	10	13.0	23.8	6	5	3	0.61	2.43	4	14	10	0.39	2.57
S0172765	10	17.1	25.0	6	8	2	0.63	2.00	4	16	10	0.40	2.53
S0175884	10	21.6	25.0	6	9	2	0.62	1.94	4	16	10	0.40	2.53
S0177499	10	25.2	25.0	6	5	2	0.65	3.15	4	12	10	0.38	2.92
S0180184	10	29.7	25.0	6	9	2	0.62	1.94	4	16	10	0.40	2.53
S0182836	10	33.7	25.0	5	9	2	0.59	1.38	4	16	12	0.38	4.00

S0188391	10	42.4	25.0	6	8	2	0.63	2.00	4	16	11	0.39	3.19
S0190237	10	46.5	26.2	6	9	2	0.62	1.94	4	17	10	0.40	2.61
S0194142	10	57.4	30.0	7	8	2	0.65	3.00	4	17	10	0.40	2.61
S0194704	10	60.4	38.2	18	25	5	0.64	7.13†	2	23	10	0.39	7.11†
S0194933	10	61.9	51.1	23	26	2	0.71	17.31†	0	24	13	0.32	12.41†
S0195109	10	63.0	62.4	33	16	2	0.80	44.76†	0	18	20	0.24	21.16†
S0195403	10	63.7	67.2	38	11	2	0.85	67.31†	0	17	21	0.22	23.63†
S0195527	10	63.9	69.6	40	9	1	0.89	81.32†	0	15	23	0.20	29.53†
S0195717	10	64.4	85.2	15	2	0	0.94	36.41†	0	0	31	0.00	93.00†
S0196053	11	0.6	0.0	7	25	18	0.39	4.84	7	22	8	0.49	1.38
S0196265	11	1.9	5.7	7	27	16	0.41	3.56	5	25	7	0.47	4.78
S0196497	11	3.6	25.8	6	27	14	0.41	3.77	4	15	17	0.32	10.39†
S0196989	11	4.6	36.1	9	24	13	0.46	0.78	5	9	21	0.27	22.89†
S0203849	11	16.5	60.7	2	9	3	0.46	1.29	3	15	13	0.34	6.48†
S0206001	11	20.5	62.9	13	26	11	0.52	0.24	5	17	13	0.39	3.69
S0213151	11	29.9	62.9	5	8	2	0.60	1.27	4	14	12	0.37	4.40
S0214214	11	32.1	62.9	4	9	2	0.57	1.13	3	14	12	0.34	5.62
S0217066	11	36.2	62.9	6	8	1	0.67	3.40	3	14	12	0.34	5.62
S0219423	11	39.2	62.9	6	6	1	0.69	3.92	4	12	12	0.36	5.14
S0220069	11	39.9	62.9	5	7	1	0.65	2.54	3	14	12	0.34	5.62
S0228219	11	48.9	64.6	6	7	4	0.56	1.00	4	14	13	0.35	5.52
S0228995	11	50.2	65.5	7	7	4	0.58	1.89	3	16	13	0.34	6.25†
S0229272	11	50.6	66.4	13	18	12	0.51	1.19	5	8	15	0.32	12.29†
S0229536	11	51.5	71.0	12	22	14	0.48	0.50	4	20	13	0.38	4.62
S0229768	11	52.1	74.9	12	28	11	0.51	0.53	5	20	12	0.41	2.89
S0229839	11	52.5	76.1	12	27	11	0.51	0.36	4	21	12	0.39	4.14
S0230640	12	0.2	0.0	15	29	5	0.60	5.73	6	16	13	0.40	3.06
S0230946	12	1.3	14.6	15	27	4	0.62	6.65†	5	20	12	0.41	2.89
S0231052	12	1.8	23.2	11	16	4	0.61	3.19	0	5	1	0.42	3.00

S0231692	12	5.8	53.7	11	21	17	0.44	2.47	11	15	10	0.51	1.06
S0231878	12	7.2	56.1	11	22	18	0.43	2.88	10	16	9	0.51	0.31
S0232145	12	8.3	56.1	11	20	18	0.43	3.65	12	13	9	0.54	2.41
S0232394	12	9.1	57.7	2	7	6	0.37	2.20	7	15	7	0.50	0.03
S0233471	12	15.2	57.7	4	7	6	0.44	1.00	8	13	7	0.52	0.21
S0238602	12	33.8	58.5	5	5	6	0.47	2.38	7	15	7	0.50	0.03
S0240093	12	40.5	59.3	3	7	6	0.41	1.38	8	14	7	0.52	0.10
S0241751	12	45.9	59.3	3	6	6	0.40	1.80	9	15	7	0.53	0.29
S0242837	12	51.5	59.3	4	6	6	0.44	1.50	9	15	6	0.55	0.60
S0243685	12	55.4	60.1	7	24	18	0.39	4.96	10	20	7	0.54	0.73
S0244678	12	58.1	60.1	3	6	6	0.40	1.80	8	11	8	0.50	0.93
S0245602	12	62.9	83.4	13	18	18	0.45	4.47	6	19	11	0.43	1.50
S0245641	12	63.4	89.3	15	19	17	0.48	3.47	6	21	11	0.43	1.74
S0245782	12	64.1	94.1	16	19	16	0.50	3.31	5	21	11	0.42	2.62
S0245979	12	65.1	101.5	16	18	13	0.53	2.96	7	20	8	0.49	0.77

† Significant deviation from 1:2:1 segregation ratio at $p < 0.05$.
