# Supplementary material for: Identification and mapping of QTLs for late blight resistance in the wild tomato (Solanum pimpinellifolium) accession PI 270442 via selective genotyping
Source: Front Plant Sci. 2024 Nov 15;15:1482241. doi: 10.3389/fpls.2024.1482241 (PMC11604435; doi:10.3389/fpls.2024.1482241)
Supplement: Supplementary file 2 [file DataSheet2.pdf]

Table S2. Candidate disease-related genes and gene models in the identified putative LB-resistance QTLs in the PI 270442; gene locations and descriptions are based on SL4.0 and ITAG4.0 annotation, respectively

Chromosome	Gene Name	Position (base)	Description
1	Solyc01g008800.3	2778190	Disease resistance protein (TIR-NBS-LRR class) (AHRD V3.3 *** F4KIC7_ARATH)
2			No known resistance proteins
3	Solyc03g053070.1	18594456	NBS-LRR resistance protein (AHRD V3.3 *-* A0A2K3MPX4_TRIPR)
3	Solyc03g065200.4	36419292	Protein ENHANCED DISEASE RESISTANCE 2-like (AHRD V3.3 *** A0A1J3E5X3_NOCCA)
3	Solyc03g078300.3	44876698	NBS-LRR resistance protein (AHRD V3.3 *** A0A2K3NU03_TRIPR)
3	Solyc03g081330.1	46760267	Avr9/Cf-9 rapidly elicited protein (AHRD V3.3 -** AT5G54165.1)
3	Solyc03g082350.2	46857748	Avr9/Cf-9 rapidly elicited protein 65 (AHRD V3.3 *** Q9FQZ0_TOBAC)
3	Solyc03g082370.1	46884935	Avr9/Cf-9 rapidly elicited protein 65 (AHRD V3.3 *** Q9FQZ0_TOBAC)
3	Solyc03g082780.3	47216403	Cf-like protein (AHRD V3.3 *** A0A140GMU4_SOLLC)
3	Solyc03g083480.4	47941558	LEAF RUST 10 DISEASE-RESISTANCE LOCUS RECEPTOR-LIKE PROTEIN KINASE-like 2.1 (AHRD V3.3 *** A0A1U8FAX0_CAPAN)
3	Solyc03g094100.1	50380280	Disease resistance protein (AHRD V3.3 *-* A0A200QRC8_9MAGN)
3	Solyc03g098740.1	55521639	Biotic cell death-associated protein (AHRD V3.3 *** Q850R9_NICGU)
3	Solyc03g114600.4	58961275	Alternaria stem canker resistance
5	Solyc05g009630.4	3872015	Disease resistance protein (AHRD V3.3 *-* A0A2U1N8H9_ARTAN),Pfam:PF18052
5	Solyc05g009740.1	3996647	Disease resistance protein (AHRD V3.3 *** A0A2U1Q201_ARTAN)
5	Solyc05g009750.1	4000905	Disease resistance protein (AHRD V3.3 *** A0A2U1N8H9_ARTAN)
5	Solyc05g009760.3	4004047	Disease resistance protein (CC-NBS-LRR class) family (AHRD V3.3 --* AT5G48620.6)
5	Solyc05g012740.2	5991664	Disease resistance protein (AHRD V3.3 *** A0A2U1N8H9_ARTAN)
5	Solyc05g012745.1	5996368	Late blight resistance protein R1-A (AHRD V3.3 *-* A0A2G2VIK4_CAPBA)
5	Solyc05g012780.4	6019719	Protein ENHANCED DISEASE RESISTANCE 2-like (AHRD V3.3 *** A0A1J3E5X3_NOCCA)
5	Solyc05g012890.1	6114357	Late blight resistance protein R1-A (AHRD V3.3 *-* A0A2G2VV43_CAPBA)
5	Solyc05g012910.4	6120522	Disease resistance protein (AHRD V3.3 *-* A0A2U1N8H9_ARTAN)
5	Solyc05g013250.3	6411571	Late blight resistance protein R1-A (AHRD V3.3 *-* A0A2G2Y9J2_CAPAN)
5	Solyc05g013280.4	6454618	Pseudomonas resistance
5			No known resistance proteins
6	Solyc06g048910.2	29619275	Disease resistance protein (AHRD V3.3 *** A0A1J3IKQ5_NOCCA)
6	Solyc06g053360.4	33901760	Protein ENHANCED DISEASE RESISTANCE 2-like (AHRD V3.3 *** A0A1J3DLC2_NOCCA)
6	Solyc06g062440.4	37045493	Disease resistance protein (AHRD V3.3 *** A0A2U1N8H9_ARTAN)
6	Solyc06g064680.1	37957967	NBS-coding resistance gene analog (AHRD V3.3 *-* B3V819_TOBAC)
6	Solyc06g064690.2	37968517	NBS-coding resistance gene analog (AHRD V3.3 *** B3V819_TOBAC)
6	Solyc06g064710.1	37975542	NB-LRR tospovirus immune receptor (AHRD V3.3 *-* A0A221IWU7_9SOLN)
6	Solyc06g064720.1	37981286	NB-LRR tospovirus immune receptor (AHRD V3.3 *** A0A221IWV0_9SOLN)
6	Solyc06g064790.1	38020792	Disease resistance protein (AHRD V3.3 *-* A0A200R7W2_9MAGN)
6	Solyc06g065120.1	38264585	Disease resistance protein (TIR-NBS-LRR class) (AHRD V3.3 *-* A0A2K3NQB9_TRIPR)

6	Solyc06g065130.1	38266573	disease resistance protein RRS1-like (AHRD V3.3 --* A0A2I4G886_9ROSI)
6	Solyc06g065140.3	38269105	Disease resistance protein (TIR-NBS-LRR class) (AHRD V3.3 *-* A0A2K3MPJ7_TRIPR)
6	Solyc06g065150.1	38270107	Disease resistance protein RPS4 (AHRD V3.3 *-* A0A1J3HFR6_NOCCA)
6	Solyc06g066490.1	39376448	Avr9/Cf-9 rapidly elicited protein 146 (AHRD V3.3 *** Q9FQZ6_TOBAC)
6	Solyc06g066500.1	39382224	Avr9/Cf-9 rapidly elicited protein 146 (AHRD V3.3 *** Q9FQZ6_TOBAC)
6	Solyc06g066510.1	39388448	Avr9/Cf-9 rapidly elicited protein 146 (AHRD V3.3 *-* Q9FQZ6_TOBAC)
6	Solyc06g066520.1	39413271	Avr9/Cf-9 rapidly elicited protein 146 (AHRD V3.3 *** Q9FQZ6_TOBAC)
6	Solyc06g071280.4	41517532	Enhanced disease susceptibility 1 protein (AHRD V3.3 *** Q2TQV0_SOLTU)
6	Solyc06g075130.4	44282107	Protein ENHANCED DISEASE RESISTANCE 2-like (AHRD V3.3 *** A0A1J3GYR1_NOCCA)
7	Solyc07g006700.1	1533538	Pathogenesis-related protein PR-1 (AHRD V3.3 *** A0A1U8H8G8_CAPAN)
7	Solyc07g006710.2	1538929	Pathogenesis-related protein PR-1 (AHRD V3.3 *** A0A2G3BX31_CAPCH)
7	Solyc07g008730.2	3678372	Pathogenesis-related homeodomain protein (AHRD V3.3 *** A0A1U8H9N2_CAPAN)
7	Solyc07g055380.1	63356441	Disease resistance protein (TIR-NBS-LRR class) (AHRD V3.3 *** A0A2K3NQB9_TRIPR)
7	Solyc07g055390.1	63360118	Disease resistance protein (TIR-NBS-LRR class) family (AHRD V3.3 *-* A0A2U1MH42_ARTAN)
7	Solyc07g055610.3	63507640	Disease resistance protein (TIR-NBS-LRR class) (AHRD V3.3 *-* A0A2K3PR54_TRIPR)
7	Solyc07g055620.2	63356441	Disease resistance protein (TIR-NBS-LRR class) (AHRD V3.3 *-* A0A2K3NQB9_TRIPR)
7	Solyc07g063360.1	65680939	Disease resistance protein (TIR-NBS-LRR class) (AHRD V3.3 *** A0A2K3MZK9_TRIPR)
10	Solyc10g047320.2	39560639	Disease resistance protein (AHRD V3.3 *** A0A2U1N8H9_ARTAN)
10	Solyc10g048040.1	42727050	Pathogenesis-related protein 1 (AHRD V3.3 *** E2GEU4_9ROSI)
10	Solyc10g048080.3	43014167	Pathogenesis-related protein 1B (AHRD V3.3 *** PR1B_TOBAC)
10	Solyc10g048100.3	43114077	Pathogenesis-related protein 1 (AHRD V3.3 *** A0A2K3L121_TRIPR)
10	Solyc10g049230.1	43657176	disease resistance protein RGA2-like (AHRD V3.3 *-* XP_010321233.1)
10	Solyc10g050050.1	46670702	Disease resistance protein (AHRD V3.3 *-* A0A200QRC8_9MAGN)
10	Solyc10g050740.1	49444274	Disease resistance protein (AHRD V3.3 *-* A0A2K3LQB5_TRIPR)
10	Solyc10g051050.3	50454520	Disease resistance protein (AHRD V3.3 *** A0A2U1N8H9_ARTAN)
10	Solyc10g051140.3	50575184	Protein ENHANCED DOWNY MILDEW 2 (AHRD V3.3 *-* A0A2G2YIJ3_CAPAN)
10	Solyc10g051150.3	50583564	protein ENHANCED DOWNY MILDEW 2-like (AHRD V3.3 *-* A0A2I4EZI8_9ROSI)
10	Solyc10g051170.2	50692812	Disease resistance protein (AHRD V3.3 *-* A0A2U1N8H9_ARTAN)
10	Solyc10g051180.1	50696857	Late blight resistance protein R1-A (AHRD V3.3 *-* R1A_SOLDE)
10	Solyc10g054350.2	54310351	Disease resistance protein (AHRD V3.3 *-* A0A2U1KZM9_ARTAN)
10	Solyc10g054360.1	54316888	NBS-LRR resistance-like protein (AHRD V3.3 *-* A0A2U1NK38_ARTAN)
10	Solyc10g054370.1	54317741	NBS-LRR resistance protein (AHRD V3.3 *-* A0A2K3P9X0_TRIPR)
10	Solyc10g054600.1	54733387	NBS-LRR resistance protein (AHRD V3.3 *-* A0A2K3NU03_TRIPR)
10	Solyc10g054610.1	54734853	NBS-LRR resistance protein (AHRD V3.3 *-* A0A2K3PMF0_TRIPR)
10	Solyc10g054940.1	55196629	Disease resistance protein (AHRD V3.3 *-* A0A200QRC8_9MAGN)
10	Solyc10g054970.1	55227374	Disease resistance protein (AHRD V3.3 *-* A0A200QRC8_9MAGN)
10	Solyc10g054990.3	55268439	disease resistance protein At4g27190-like (AHRD V3.3 *-* A0A2I4EJA4_9ROSI)
10	Solyc10g055050.2	55343562	Disease resistance protein (AHRD V3.3 *-* A0A2U1P003_ARTAN)

10	Solyc10g055170.1	55489536	Disease resistance protein (AHRD V3.3 *-* A0A200QUI0_9MAGN)
10	Solyc10g074640.3	57198963	Disease resistance protein (AHRD V3.3 *-* A0A200QUG5_9MAGN)
10	Solyc10g076440.1	58511373	Disease resistance protein (AHRD V3.3 *** A0A2U1P5P8_ARTAN)
10	Solyc10g084880.3	63412965	Avr9/Cf-9 rapidly elicited protein 137 (AHRD V3.3 *** Q9FQZ2_TOBAC)
11	Solyc11g011080.3	4194735	Disease resistance protein (TIR-NBS-LRR class) (AHRD V3.3 *** A0A2K3NNZ4_TRIPR)
11	Solyc11g011090.1	4200433	Disease resistance protein (TIR-NBS-LRR class) (AHRD V3.3 *** A0A2K3P9P3_TRIPR)
11	Solyc11g011350.3	4422453	Disease resistance protein (TIR-NBS-LRR class) (AHRD V3.3 *** A0A2K3MNP1_TRIPR)
11	Solyc11g013750.3	7170382	Disease resistance protein (TIR-NBS-LRR class) family (AHRD V3.3 *-* A0A1P8BBG0_ARATH)
11	Solyc11g020080.3	10226288	Disease resistance protein (AHRD V3.3 *-* A0A2U1N8H9_ARTAN)
11	Solyc11g020090.1	10226935	NBS-LRR protein (AHRD V3.3 *-* Q75UQ0_IPOBA)
11	Solyc11g020100.3	10229943	Disease resistance protein (AHRD V3.3 *** A0A2U1N8H9_ARTAN)
11	Solyc11g045410.1	29387487	CC-NBS-LRR type resistance-like protein (AHRD V3.3 *-* E3W9R6_CAPCH)
11	Solyc11g044400.1	31512307	Pathogenesis-related thaumatin family protein (AHRD V3.3 *-* A0A2K3LJG6_TRIPR)
11	Solyc11g043100.1	32381974	Disease resistance protein R3a-like protein (AHRD V3.3 *-* Q6L3G6_SOLDE)
11	Solyc11g043090.1	32383568	CC-NBS-LRR type resistance-like protein (AHRD V3.3 *-* E3W9R6_CAPCH)
11	Solyc11g043070.3	32436916	CC-NBS-LRR type resistance protein (AHRD V3.3 *-* E3W9R7_CAPAN)
11	Solyc11g043060.1	32439153	CC-NBS-LRR type resistance protein (AHRD V3.3 *-* E3W9S0_CAPFR)
11	Solyc11g043030.1	32469588	CC-NBS-LRR type resistance protein (AHRD V3.3 *-* E3W9R7_CAPAN)
11	Solyc11g042770.1	33447792	CC-NBS-LRR type resistance protein (AHRD V3.3 *-* E3W9R9_CAPCH)
11	Solyc11g042750.1	33483844	CC-NBS-LRR type resistance protein (AHRD V3.3 *-* E3W9R5_CAPCH)
11	Solyc11g042730.3	33492040	CC-NBS-LRR type resistance protein (AHRD V3.3 *-* E3W9R9_CAPCH)
11	Solyc11g042720.1	33492501	CC-NBS-LRR type resistance-like protein (AHRD V3.3 *-* E3W9R6_CAPCH)
11	Solyc11g062150.3	46961662	Disease resistance protein (TIR-NBS-LRR class) family (AHRD V3.3 *-* A0A2U1P7P1_ARTAN)
11	Solyc11g062180.1	46983996	Disease resistance protein (TIR-NBS-LRR class) (AHRD V3.3 *-* A0A2K3N2V7_TRIPR)
11	Solyc11g064760.1	47948964	CC-NBS-LRR type resistance protein (AHRD V3.3 *-* E3W9R7_CAPAN)
11	Solyc11g064770.3	47949387	CC-NBS-LRR type resistance protein (AHRD V3.3 *** E3W9R5_CAPCH)
11	Solyc11g065780.3	49432510	CC-NBS-LRR type resistance-like protein (AHRD V3.3 *-* E3W9R6_CAPCH)
11	Solyc11g065790.1	49433095	Disease resistance protein I2 (AHRD V3.3 *-* Q9XET3_SOLLC)
11	Solyc11g065810.1	49435069	CC-NBS-LRR type resistance protein (AHRD V3.3 *-* E3W9S0_CAPFR)
12	Solyc12g005520.1	312150	Disease resistance protein (CC-NBS-LRR class) family (AHRD V3.3 --* AT1G12290.4)
12	Solyc12g005530.1	312554	Disease resistance protein (AHRD V3.3 *-* A0A2U1P5P8_ARTAN)
12	Solyc12g005540.1	312994	Disease resistance protein (AHRD V3.3 --* A0A2U1P5P8_ARTAN)
12	Solyc12g005970.1	605768	NBS-LRR protein (AHRD V3.3 *-* Q9SM52_SOLAC)
12	Solyc12g006040.3	643740	NBS-LRR protein (AHRD V3.3 *-* Q9SM52_SOLAC)
12			No known resistance proteins
